# A Concise Synthetic Method for Constructing 3-Substituted Piperazine-2-Acetic Acid Esters from 1,2-Diamines

**DOI:** 10.3390/molecules27113419

**Published:** 2022-05-25

**Authors:** Srinivas Chamakuri, Sunny Ann Tang, Kevin A. Tran, Shiva Krishna Reddy Guduru, Peter K. Bolin, Kevin R. MacKenzie, Damian W. Young

**Affiliations:** 1 Center for Drug Discovery, Department of Pathology and Immunology, Baylor College of Medicine, One Baylor Plaza, Houston, TX 77030, USA; sunny.tang@bcm.edu (S.A.T.); kevin.tran@bcm.edu (K.A.T.); shiva.guduru@bcm.edu (S.K.R.G.); peter.bolin@bcm.edu (P.K.B.); kevin.mackenzie@bcm.edu (K.R.M.); 2 Department of Pharmacology and Chemical Biology, Baylor College of Medicine, One Baylor Plaza, Houston, TX 77030, USA; 3 Verna and Marrs McLean Department of Biochemistry and Molecular Biology, Baylor College of Medicine, Houston, TX 77030, USA

**Keywords:** chiral piperazines, 2,3-substituted piperazines, diamine, annulation, masamune condensation

## Abstract

We report a short synthetic route for synthesizing 2,3-substituted piperazine acetic acid esters. Optically pure amino acids were efficiently converted into 1,2-diamines that could be utilized to deliver the title 2,3-substituted piperazines in five steps with a high enantiomeric purity. The novel route facilitated, for the first time, the synthesis of 3-phenyl substituted-2-piperazine acetic acid esters that were difficult to achieve using other methods; however, in this case, the products underwent racemization.

## 1. Introduction

The piperazine ring constitutes an important class of saturated nitrogen-containing heterocycles and is the third most common ring system found in FDA approved drugs [1,2,3,4,5,6]. Interestingly, however, more than 80% of these compounds are devoid of substitutions on the methylene carbon atoms of the piperazine ring. Given their tetrahedral geometry, the lack of structural diversity involving the piperazine carbon atoms represent an untapped potential for expanding the 3-dimensional chemical space for improving molecular recognition toward a spectrum of biological targets [7,8,9,10,11,12,13,14,15]. This encourages the development of new, efficient, and selective methods for accessing the carbon functionalization of the piperazine ring. Toward achieving this goal, we have previously reported the synthesis of a family of chiral disubstituted piperazine-2-acetic acid esters. Starting from amino acids (natural and unnatural), we constructed 3-, 5- and 6- substituted piperazine-2-acetic acid esters comprising both relative and absolute stereoisomers as single compounds [16,17,18,19,20].

**Scheme 1:**
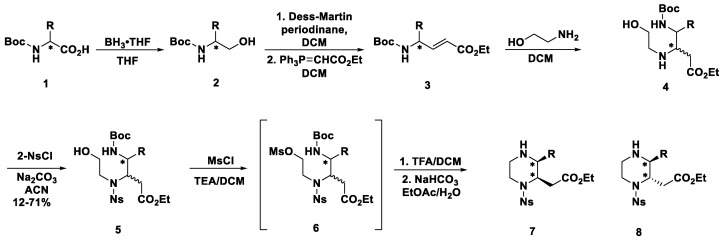
Previous synthesis of 3-substituted piperazine-2-acetic acid esters; * = enantio pure; R = Me, Bn, *^i^*Bu, *^i^*Pr, CH_2_OBn; Ns = 2-nitrobenzenesulfonyl.

During the course of our studies of the 2,3-substituted piperazine series, we noticed that despite the prevalence of vicinal substituted 1,2-diamines, there were relatively few methods aimed at generating a range of 2,3-substituted piperazines [21,22]. We developed a route to 2,3-substituted piperazines using an intermolecular aza Michael reaction on α, β-unsaturated esters (3 to 4 in Scheme 1) followed by a terminal intramolecular S_N_2 ring closure (6 to 7 in Scheme 1) to furnish the piperazine core. This seven step sequence led to the production of optically pure quantities of 3-substituted-2-piperazine acetic acid esters on a gram scale; however, a major limitation in the methodology was that we were unable to access piperazine products containing a phenyl substitution at the 3-position via this route. This prompted further investigation toward preparing these compounds, resulting in a more efficient synthesis of 3-substituted piperazine-2-acetic acid esters that we report herein.

## 2. Results and Discussion

**Scheme 2:**
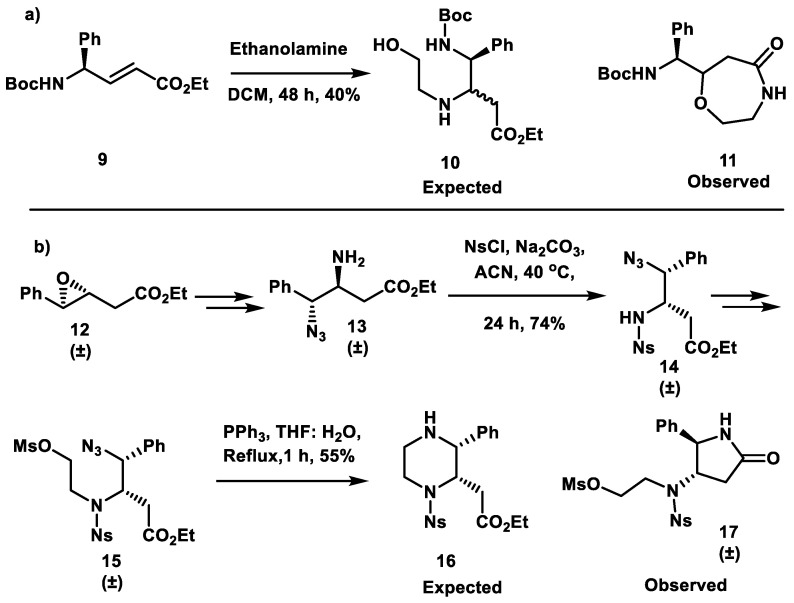
Failure attempts to the synthesis of 3-substituted piperazine-2-acetic acid esters; (**a**) Michael addition of ethanolamine to the α, β-unsaturated ester 9; (**b**) late stage intramolecular S_N_2 ring closure to obtain 16; Ns = 2-nitrobenzenesulfonyl.

Phenyl substituted piperazines are substrates with precedent biological activity inciting our interest in the more stereo-chemically diverse 2,3-disubstituted series [23,24,25,26]. In prior studies [17], we applied our optimized synthetic route, which entailed the generation of the key aza Michael precursor **9**. However, upon the treatment of ethanolamine, instead of generating the desired piperazine precursor **10**, the seven-membered lactam **11** ring was observed as the major product (Scheme 2a). We further attempted the reaction using more reactive Michael acceptors (α, β-unsaturated nitrile, not shown), but this did not result in the desired conjugate addition product. We rationalized that steric hindrance arising from both -NHBoc and -Ph groups was hampering the Michael addition of the ethanolamine. The failed attempts to furnish the 1,4-diamine through aza Michael reaction led us to pursue an alternative route involving the preparation of azidoamine **13** [27]. We imagined that this precursor could be delivered from epoxide **12** through several functional group transformations. Indeed, the alkylation of **14** with a protected iodoethanol, followed by conversion of the alcohol to the mesylate, generated the key piperazine precursor **15**. However, rather than a conversion to the piperazine, the subjection of the azide to the Staudinger conditions resulted in a ring closure of the five-membered lactam (Scheme 2b). These failed attempts highlight the difficulty in controlling reactivity in densely functionalized molecules.

**Scheme 3:**
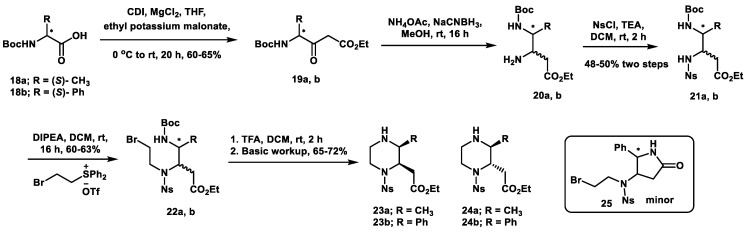
Synthesis of 3-substituted piperazine-2-acetic acid esters; * = enantio pure; Ns = 2-nitrobenzenesulfonyl or 4-nitrobenzenesulfonyl.

We next wondered whether alternative annulation conditions would encourage piperazine formation over lactam formation. We previously reported a practical route to differentially protected 2-substituted piperazines through the reaction of 1,4-diamines with vinyl diphenyl phosponium reagents [19], and next sought to apply this methodology to the 2,3-disubstituted piperazines. We planned a route to generate the key intermediate 2,3-substituted 1,4-diamine from the β-keto ester **19** that could be obtained from an amino acid (Scheme 3) [28]. To test this route, we started from *N*-Boc protected (*S*)-alanine **18a** and converted it to the β-ketoester (**19a**) using a Masamune condensation reaction. Next, the reductive amination of **19a** with ammonium acetate and sodium cyanoborohydride yielded the 2,3-substituted 1,4-diamine **20a** as a ~1:1 diasteromeric mixture (from crude NMR) in a good yield [29]. The newly generated amine was protected using 2-NsCl to afford **21a** as the key piperazine ring precursor. Subjection to the annulation protocol by treatment with bromoethyldiphenylsulfonium triflate yielded **22a**, which gratifyingly underwent cyclization on Boc removal with TFA followed by a basic workup to obtain the diastereomeric products (*cis* and *trans*) **23a** and **24a** as a ~1:1 mixture. The diastereomeric piperazines were efficiently separated using column chromatography and characterized by ^1^H and ^13^C NMR. The final piperazine compounds protected with Boc on N_1_ (by following literature protocol, see the details in Supplementary Materials) were compared with our previous report, and we tested the chiral HPLC and confirmed that no erosion of the stereo-isomeric purity occurred during this new synthetic process. 

With this new method, we turned our attention to synthesizing phenyl-substituted piperazine starting from chiral phenylglycine amino acid. Accordingly, the β-keto ester obtained from phenylglycine was subjected to reductive amination to achieve **20b**, which was in turn nosylated to achieve key intermediate **21b**. We noted that in this instance, attempts using 2-NsCl were unsuccessful, but using 4-NsCl was successful. The orthogonally protected diamine **21b** was treated with bromoethyldiphenylsulfonium triflate followed by TFA deprotection to furnish the expected piperazine products **23b** and **24b** in a 2:1 ratio. We also observed a small amount of the five-membered lactam (**25**, by LCMS) during the cyclization reaction. Fortunately, this side product was avoided by performing the neutralization at a lower temperature (0 °C) after the Boc removal (see the experimental procedure for details). With the final phenyl substituted piperazine products in hand, we once again checked the enantiomeric excess using a chiral HPLC. Unfortunately, in this case, we discovered both enantiomers in a 1:1 ratio. Even though the conditions were mild, we imagined that racemization was occurring during the first step to form the β-ketoester from phenylglycine. To confirm this, we attempted the formation of **19b** using both racemic and enantiomerically pure *S*-phenylglycine amino acids. We found that the chiral *S*-phenylglycine afforded the same racemic mixture as the racemic phenylglycine, as evidenced by chiral HPLC (see Supplementary Materials).

**Scheme 4:**
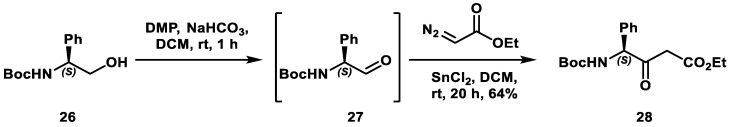
Synthesis of chiral β-ketoester 28.

To address the racemization issue, we hypothesized that bypassing an activated carboxylic acid to obtain the β-ketoester intermediate would eliminate the propensity for enolization. We adopted the method of Mothukuri et al. to generate β-ketoesters from phenylglycinal (**27**) by treating it with ethyl diazoacetate in the presence of the Lewis acid SnCl_2_ [30,31]. Starting from the optically pure S-phenylgylcinol, a Dess–Martin oxidation was performed to generate **27**, which was then submitted to the procedure advanced by Mothukuri et al. to afford **28** in a 64% yield (Scheme 4) [30]. To our delight, we confirmed that **28** was formed in greater than >99% ee (see Supplementary Materials). We then proceeded to transform the enantiopure β-ketoester (**28**) into the final products (**23b** and **24b**) using the previously described synthetic route (Scheme 3). However, our final validation of the stereo-chemical purity was met with disappointment, given that the phenyl piperazine products had completely racemized. Undoubtedly, the loss of stereo-chemical integrity in this instance was occurring during the reductive amination reaction. Further studies are warranted toward the generation of optically pure phenyl piperazine. Nonetheless, these studies have established, for the first time, a route to 3-phenyl substituted piperazine-2-acetic acid esters.

In conclusion, we have developed an efficient five-step synthetic route toward generation of 3-substituted piperazine-2-acetic acid esters starting from amino acids. The developed route features the synthesis of a key chiral 1,2-diamine intermediate that undergoes annulation to the desired enantiopure 3-substituted piperazine-2-acetic acid esters. The route was successfully applied to the synthesis of 3-phenyl piperazine-2-acetic acid ester, which proved elusive using previously developed methodologies; however, the final products underwent racemization. These efforts add to the synthetic armamentarium for producing novel biologically valuable piperazine heterocycles.

## 3. Experimental

All starting materials and reagents were purchased from commercial sources and used without further purification. Solvents were purchased as either anhydrous grade products in sealed containers or reagent grade and used as received. All reactions were carried out in dry glassware under a nitrogen atmosphere using standard disposable or gastight syringes, disposable or stainless steel needles, and septa. Stirring was achieved with magnetic stir bars. Flash column chromatography was performed with SiO_2_ (230–400 mesh), or by using an automated chromatography instrument with an appropriately sized column. Thin layer chromatography was performed on silica gel 60F_254_ plates (E. Merck). Non-UV active compounds were visualized on TLC using one of the following stains: KMnO_4_, ninhydrin, and *p*-anisaldehyde. ^1^H and ^13^C NMR spectra were recorded on an instrument operating at both 600 MHz and 151 MHz, respectively. LCMS data were collected using an HPLC instrument coupled to a low-resolution mass spectrometer with single quadrupole ionization operating in either a positive or negative ion mode. The analytical method utilized a C_18_ column (2.1 × 50 mm, 1.8 µm) eluting with a linear gradient of 95%/5% water/CH_3_CN (modified with 0.05% formic acid; T = 0 min flow = 0.35 mL/min) to 95%/5% CH_3_CN/water (T = 3.5 min flow = 0.5 mL/min) then 95%/5% CH_3_CN/water to T = 5 min (0.5 mL/min). Peak detection was done at 254 nm and 230 nm for UV active compounds. For non-UV active compounds, a total ion count was used. The Chiral HPLC analysis of piperazines was carried out on an instrument with automated six-column array (Daicel ChiralPak I-series, IA through IF, 4.6 × 150 mm, 5 µm). The racemates were screened using a heptane (A)/ethanol (B) gradient (flow = 1 mL/min) as follows: T = 0 min (%A/%B) 95/5, T = 1 min 95/5, T = 11 min 10/90 (linear gradient), T = 13 min 10/90, T = 13.1 min 95/5, and T = 15 min 95/5. Racemates that provided us with an unsatisfactory resolution of the enantiomers were rescreened using the above gradient with iPrOH instead of EtOH. Additional editing of the gradient, or the use of an isocratic mobile phase to optimize separation, was carried out as needed. All NMR chemical shifts are quoted on the δ scale and were referenced to residual non-deuterated solvent as an internal standard. Signal multiplicities are described using the following abbreviations: s = singlet, d = doublet, t = triplet, b = broad, quar = quartet, quin = quintet, m = multiplet, v = very; abbreviations are combined, e.g., vbs = very broad singlet, FWHM = width at half maximum.

General Procedure for 19a, b: Into a round-bottomed flask equipped with magnetic stir bar and septum, Boc-protected amino acid (1 equiv.) was dissolved in anhydrous THF and allowed to cool to °C. Next, carbonyldiimidazole (CDI) (1.25 equiv.) was added (under nitrogen atmosphere) and the solution was stirred for 2 h at the same temperature. Magnesium chloride (1.2 equiv.) and ethyl potassium malonate (1.5 equiv.) were added and the reaction (flushed with nitrogen) was stirred at room temperature for 16 h, after which time the TLC and LCMS showed no starting material remaining. The reaction mixture was evaporated to remove THF; it was then dissolved in ethyl acetate and washed with water and brine. The organic phase was collected and dried over anhydrous Na_2_SO_4_. The solvent was removed under reduced pressure to obtain the crude residue. Purification by silica gel chromatography (ethyl acetate/hexanes) provided the pure bromide product.

Ethyl (S)-4-((tert-butoxycarbonyl)amino)-3-oxopentanoate (**19a**); Formula: C_12_H_21_NO_5_; ^1^H NMR (600 MHz, CDCl_3_) δ 5.14–5.12 (m, 1H), 4.40–4.37 (m, 1H), 4.20 (q, *J* = 7.1 Hz, 2H), 3.55 (q, *J* = 15.8 Hz, 2H), 1.44 (s, 9H), 1.36 (d, *J* = 7.1 Hz, 3H), 1.28 (t, *J* = 7.1 Hz, 3H). ^13^C NMR (151 MHz, CDCl_3_) δ 202.4, 166.9, 155.2, 80.1, 61.5, 60.3, 55.4, 45.9, 28.3, 19.3, 17.1, 14.1.

Ethyl (S)-4-((tert-butoxycarbonyl)amino)-3-oxo-4-phenylbutanoate (**28**); Formula: C_17_H_23_NO_5_; ^1^H NMR (600 MHz, CDCl_3_) δ 7.39–7.30 (m, 5H), 5.83 (d, *J* = 6.4 Hz, 1H), 5.45 (d, *J* = 6.5 Hz, 2H), 4.13–4.09 (m, 2H), 3.47 (d, *J* = 15.8 Hz, 1H), 3.32 (d, *J* = 15.8 Hz, 1H), 1.41 (s, 9H), 1.22 (t, *J* = 7.2 Hz, 3H). ^13^C NMR (151 MHz, CDCl_3_) δ 198.4, 166.2, 154.7, 129.3, 128.8, 128.1, 127.1, 80.1, 64.4, 61.6, 46.1, 28.3, 14.0.

General Procedure for 20a-b: Into a round-bottomed flask equipped with magnetic stir bar and septum, the β-ketoester (1 equiv.) was dissolved in dry MeOH, and then NH_4_OAc (20 equiv.) was added. The solution was stirred at room temperature for 10 min, then NaBH_3_CN (3.5 equiv.) was added in one portion and the resulting mixture was stirred at room temperature for 20 h, after which time the TLC and LCMS showed no starting material remaining. The reaction was quenched with 1 N HCl. The solvent was evaporated and the crude residue was dissolved in water and neutralized with an aq. NaHCO_3_ solution. The aqueous phase was extracted with ethyl acetate and washed with brine. The organic phase was collected and dried over anhydrous Na_2_SO_4_. The solvent was removed under reduced pressure to obtain the crude residue. The crude product was further subjected to the next reaction without purification.

General Procedure for Nosylation (sulfonylation) 21a, b: Into a round bottom flask equipped with magnetic stir bar and septum, the Boc-protected amine (1 equiv.) was dissolved in dichloromethane, and TEA (2 equiv.) was added, followed by 2-nitrobenzene sulfonylchloride (2-nosylchloride, 1.2 equiv.). The reaction was stirred at room temperature for 2 h under nitrogen, after which time the TLC and LCMS indicated the complete consumption of the starting material. The reaction was diluted with dichloromethane and washed with a saturated aq NaHCO_3_ and brine solution. The organic phase was collected and dried over anhydrous Na_2_SO_4_ and the solvent was removed under reduced pressure to give the crude residue. Purification by silica gel chromatography (ethyl acetate/hexanes) provided the pure product (mixture of diastereomers).

General Procedure for 22a, b: Into a round bottom flask equipped with magnetic stir bar and septum under nitrogen, the bisprotected chiral diamine (1 equiv.) was dissolved in dichloromethane, and 2-bromoethyl-diphenylsulfonium triflate (1.25 equiv.) was added followed by diisopropylethylamine (3 equiv.). The reaction was allowed to stir at room temperature under nitrogen for 16 h, after which time the TLC and LCMS showed no starting material remaining. The reaction was generated by diluting with dichloromethane and washing with water and brine. The organic phase was collected and dried over anhydrous Na_2_SO_4_. The solvent was removed under reduced pressure to obtain the crude residue. Purification by silica gel chromatography (ethyl acetate/hexanes) provided the pure bromide product (mixture of diastereomers).

General Procedure for 23 & 24 a, b: Into a round bottom flask equipped with magnetic stir bar and septum, the bromide compound (1 equiv.) was dissolved in dichloromethane, and TFA (10% TFA in DCM, *v*/*v*, 10 mL/mmol) was added and allowed to stir at room temperature under nitrogen. After 2 h, the starting material was consumed according to the TLC and LCMS. The volatiles were evaporated under reduced pressure and then redissolved in DCM and then cooled to 0 °C and neutralized with a saturated aq NaHCO_3_ solution. The organic phase was collected and dried over anhydrous Na_2_SO_4_ and the solvent was removed under reduced pressure to obtain the crude residue. Purification by silica gel chromatography (ethyl acetate/hexanes) provided the pure piperazine products.

Note; 23a and 24a are further subjected to Boc-protection on N_1_ and compared with literature compounds (see Supplementary Materials).

Ethyl 2-(1-((4-nitrophenyl)sulfonyl)-3-phenylpiperazin-2-yl)acetate (*cis*, **23b**); Formula: C_20_H_23_N_3_O_6_S; ^1^H NMR (600 MHz, CDCl_3_) δ 8.27 (d, *J* = 8.9 Hz, 2H), 7.99 (d, *J* = 8.9 Hz, 2H), 7.27–7.22 (m, 4H), 7.20 (d, *J* = 6.6 Hz, 1H), 4.61–4.58 (m, 1H), 3.95 (d, *J* = 3.5 Hz, 1H), 3.76–3.73 (m, 1H), 3.62 (q, *J* = 7.1 Hz, 2H), 3.19–3.14 (m, 1H), 3.07–3.04 (m, 1H), 2.91–2.86 (m, 1H), 2.41–2.33 (m, 2H), 0.96 (t, *J* = 7.1 Hz, 3H). ^13^C NMR (151 MHz, CDCl_3_) δ 170.7, 149.9, 147.0, 139.0, 128.7, 128.4, 128.1, 126.8, 124.3, 62.9, 60.5, 56.0, 46.5, 41.1, 30.9, 13.9.

Ethyl 2-(1-((4-nitrophenyl)sulfonyl)-3-phenylpiperazin-2-yl)acetate (*trans*, **24b**); Formula: C_20_H_23_N_3_O_6_S; ^1^H NMR (600 MHz, CDCl_3_) δ 8.11 (d, *J* = 8.8 Hz, 2H), 7.70 (d, *J* = 8.8 Hz, 2H), 7.36–7.34 (m, 2H), 7.27–7.25 (m, 3H), 4.76–4.74 (m, 1H), 4.17–4.11 (m, 2H), 4.03 (d, *J* = 2.6 Hz, 1H), 3.59–3.56 (m, 1H), 3.34–3.30 (m, 1H), 3.25 (dd, *J* = 16.2, 9.5 Hz, 1H), 3.02 (td, *J* = 11.8, 4.1 Hz, 1H), 2.88–2.85 (m, 1H), 2.61 (dd, *J* = 16.2, 3.7 Hz, 1H), 1.26 (t, *J* = 7.1 Hz, 3H). ^13^C NMR (151 MHz, CDCl_3_) δ 171.0, 149.8, 145.7, 139.9, 128.7, 128.2, 127.3, 126.9, 124.1, 61.0, 57.1, 54.6, 41.6, 40.0, 36.1, 14.2.

## Data Availability

Data is contained within the article or Supplementary Materials.

## Supplementary Materials

The following supporting information can be downloaded at: https://www.mdpi.com/article/10.3390/molecules27113419/s1.

## Sample Availability

Samples of the compounds and key intermediates are available from the authors.
