# Supplementary material for: A Concise Synthetic Method for Constructing 3-Substituted Piperazine-2-Acetic Acid Esters from 1,2-Diamines
Source: Molecules. 2022 May 25;27(11):3419. doi: 10.3390/molecules27113419 (PMC9182393; doi:10.3390/molecules27113419)
Supplement: Supplementary file 1 [file molecules-27-03419-s001.zip › molecules-1722859-supplementary.pdf]

Supplementary Material

A Concise Synthetic Method for Constructing 3-Substituted Piperazine-2-Acetic Acid Esters from 1,2-Diamines

Srinivas Chamakuri [1,*], Sunny Ann Tang [1], Kevin A. Tran [1], Shiva Krishna Reddy Guduru [1], Peter K. Bolin [1], Kevin R. MacKenzie [1,2] and Damian W. Young [1,2,3,*]

[1] Center for Drug Discovery, Department of Pathology and Immunology, Baylor College of Medicine, One Baylor Plaza, Houston, TX 77030, USA; sunny.tang@bcm.edu (S.A.T.); kevin.tran@bcm.edu (K.A.T.); shiva.guduru@bcm.edu (S.K.R.G.); peter.bolin@bcm.edu (P.K.B.); kevin.mackenzie@bcm.edu (K.R.M.)

[2] Department of Pharmacology and Chemical Biology, Baylor College of Medicine, One Baylor Plaza,
Houston, TX 77030, USA

[3] Verna and Marrs McLean Department of Biochemistry and Molecular Biology, Baylor College of Medicine, Houston, TX 77030, USA

* Correspondence: srinivas.chamakuri@bcm.edu (S.C.); damian.young@bcm.edu (D.W.Y.)

Supporting Information
Table of Contents

Content	Page
Experimental procedures, NMR data and NMR assignments	S2-S5
^{1}H, ^{13}C NMR spectra of all new compounds	S6-S30
Chiral HPLC data	S31-S44

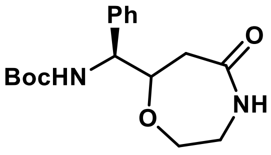

11

Tert-butyl ((1S)-(5-oxo-1,4-oxazepan-7-yl)(phenyl)methyl)carbamate (11): Into a round-bottomed flask equipped with magnetic stir bar and septum, *α*, β- unsaturated olefin (**1 equiv.**) was dissolved in anhydrous DCM. Next, ethanolamine (**4 equiv.**) was added by syringe. The homogeneous solution was stirred at rt for 48h, after which time the TLC and LCMS showed no starting material remaining. The reaction mixture was evaporated to an oil, redissolved in ethyl acetate and washed with water and brine. The organic phase was collected and dried over anhydrous Na_2SO_4. The solvent was removed under reduced pressure to give the crude residue. Purification by silica gel chromatography (ethyl acetate/ hexanes) provided the pure product 11. Yield; 40%. Formula: $C_{17}H_{24}N_2O_4$; **^{1}H NMR (600 MHz, $CDCl_3$)** δ 7.43-7.41 (m, 2H), 7.38 – 7.31 (m, 3H), 7.03 (bs, 1H), 5.84 (bs, 1H), 3.73 – 3.66 (m, 2H), 3.45 (bs, 1H), 3.42 – 3.34 (m, 2H), 3.19 – 3.17 (m, 2H), 1.49-1.47 (m, 9H).

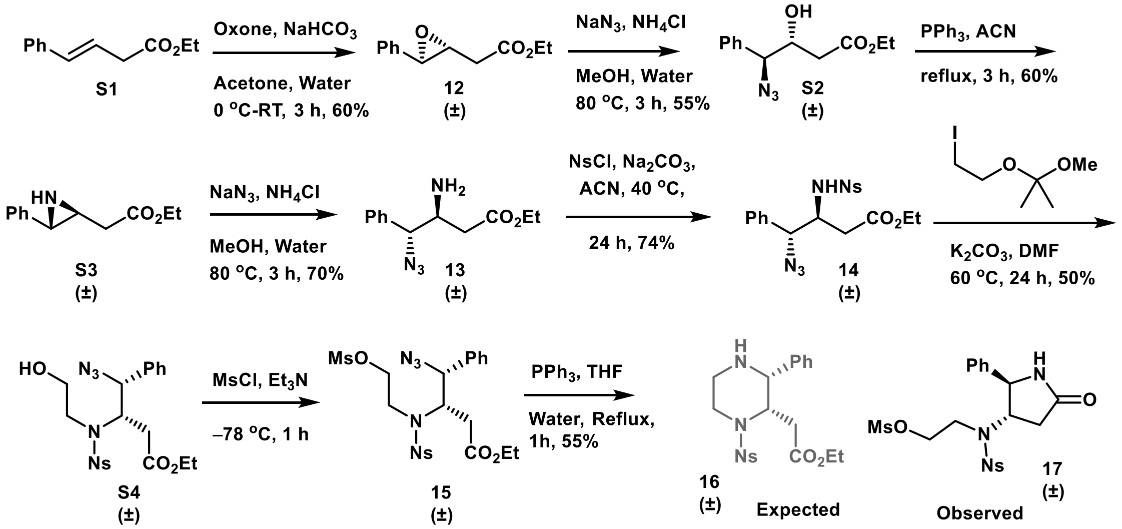

Scheme S1: synthesis of 3-Phenyl substituted piperazines-2-acetic acid ester

The following compounds synthesized by following literature procedure [1 & 2].

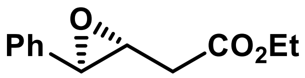

12
(±)

Ethyl 2-(3-phenyloxiran-2-yl)acetate (12); Formula: $C_{12}H_{14}O_3$; **^{1}H NMR (600 MHz, $CDCl_3$)** δ 7.39 – 7.30 (m, 5H), 4.22 (qd, *J* = 7.1, 1.9 Hz, 2H), 3.74 (d, *J* = 2.1 Hz, 1H), 3.37 (ddd, *J* = 6.2, 5.2, 2.1 Hz, 1H), 2.79 – 2.69 (m, 2H), 1.31 (t, *J* = 7.2 Hz, 3H).

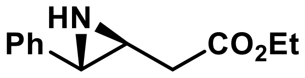

S3
(±)

Ethyl 2-(3-phenylaziridin-2-yl)acetate; Formula: $C_{12}H_{15}NO_2$; **^{1}H NMR (600 MHz, $CDCl_3$)** δ 7.35 – 7.32 (m, 2H), 7.28 – 7.25 (m, 1H), 7.24 – 7.22 (m, 2H), 4.20 (qd, *J* = 7.1, 1.4 Hz, 2H), 2.81 – 2.79 (m, 1H), 2.69 – 2.62 (m, 1H), 2.57-2.53 (m, 2H), 1.30 (t, *J* = 7.2 Hz, 3H).

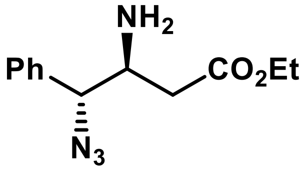

13
(±)

Ethyl (3S,4R)-3-amino-4-azido-4-phenylbutanoate; Formula: $C_{12}H_{16}N_4O_2$; **^{1}H NMR (600 MHz, $CDCl_3$)** δ 7.46 – 7.44 (m, 2H), 7.41 – 7.39 (m, 3H), 4.81 – 4.74 (m, 1H), 4.17 (q, *J* = 7.1 Hz, 2H), 3.60 – 3.57 (m, 1H), 2.68 (dd, *J* = 16.5, 3.8 Hz, 1H), 2.55 – 2.51 (m, 1H), 1.28 (t, *J* = 7.1, 3H).

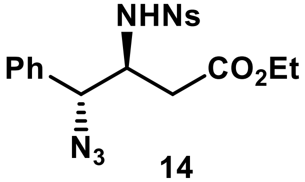

14
(±)

Ethyl (3S,4R)-4-azido-3-((4-nitrophenyl)sulfonamido)-4-phenylbutanoate; Formula: $C_{18}H_{19}N_5O_6S$; **^{1}H NMR (600 MHz, $CDCl_3$)** δ 8.01 – 7.99 (m, 1H), 7.86 – 7.84 (m, 1H), 7.71 – 7.67 (m, 2H), 7.28 – 7.24 (m, 5H), 6.12 (d, *J* = 8.5 Hz, 1H), 4.93 (d, *J* = 6.3 Hz, 1H), 4.15-4.10 (m, 1H), 4.05 (q, *J* = 7.1 Hz, 2H), 2.72 (dd, *J* = 16.6, 6.3 Hz, 1H), 2.52 (dd, *J* = 16.6, 4.8 Hz, 1H), 1.23 (t, *J* = 7.1, 3H).

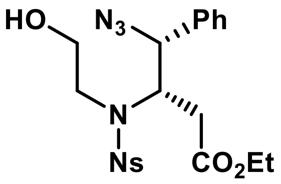

S4
(±)

Ethyl (3S,4R)-4-azido-3-((N-(2-hydroxyethyl)-4-nitrophenyl)sulfonamido)-4-phenylbutanoate; Formula: $C_{20}H_{23}N_5O_7S$; **^{1}H NMR (600 MHz, $CDCl_3$)** δ 8.05 (dd, *J* = 7.9, 1.5 Hz, 1H), 7.72 (td, *J* = 7.6, 1.4 Hz, 1H), 7.68 (td, *J* = 7.7, 1.4 Hz, 1H), 7.60 (dd, *J* = 7.8, 1.4 Hz, 1H), 7.35-7.31 (m, 5H), 5.10 (d, *J* = 5.7 Hz, 1H), 4.43-4.40 (m, 1H), 3.96 (q, *J* = 7.1 Hz, 2H), 3.88 (ddd, *J* = 11.5, 6.9, 4.3 Hz, 1H), 3.83 – 3.79 (m, 1H), 3.72 (ddd, *J* = 15.7, 6.9, 4.3 Hz, 1H), 3.43-3.40 (m, 1H), 2.90 (dd, *J* = 16.4, 8.3 Hz, 1H), 2.75 (dd, *J* = 16.4, 5.0 Hz, 1H), 1.19 (t, *J* = 7.2 Hz, 3H).

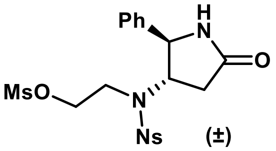

(±)
17

Note: Crude NMR;

2-((4-nitro-N-((2R,3S)-5-oxo-2-phenylpyrrolidin-3-yl)phenyl)sulfonamido)ethyl methanesulfonate; Formula: $C_{19}H_{21}N_3O_8S_2$; **^{1}H NMR (600 MHz, $CDCl_3$)** δ 7.99 (dd, *J* = 7.9, 1.3 Hz, 1H), 7.76 (ddd, *J* = 8.0, 7.4, 1.4 Hz, 1H), 7.68 – 7.64 (m, 1H), 7.38 – 7.32 (m, 3H), 7.31 – 7.26 (m, 4H), 5.58 (d, *J* = 4.3 Hz, 1H), 4.72 (ddd, *J* = 8.8, 5.2, 4.3 Hz, 1H), 4.46 (t, *J* = 5.6 Hz, 2H), 3.81-3.77 (m, 2H), 3.07 (s, 3H), 3.02 – 2.95 (m, 1H), 2.76 – 2.69 (m, 1H).

23a and 24a are further subjected to Boc-protection on N_1 and compared with literature compounds [1].

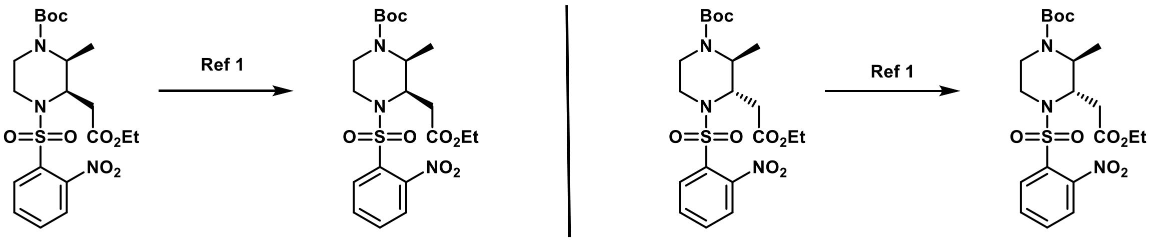

23a **S5** **23a** **S6**

Analyzing NMR data for the 23b and 24b piperazine products

Assignments for a single set of resonances were unambiguously inferred for **23b** and **24b** in deuterated chloroform from 2D HSQC and HMBC spectra (Table 1).

The expected ^{1}H-^{1}H J couplings for six membered rings in a canonical chair conformation are: ^2J ~12-15 Hz; $^3J_{ax\text{-}ax}$ 12-13 Hz, $^3J_{ax\text{-}eq}$ ~3.5-4.0 Hz, $^3J_{eq\text{-}eq}$ < 2 Hz.

^{3}J analysis for 23b. H2 (4.59 ppm) is a multiplet due to the ester CH_2, but splitting of H3 (3.95 ppm, 3.2 Hz doublet) matches $^3J_{ax\text{-}eq}$. H6 (3.74 ppm, 13.5 Hz doublet, <2Hz multiplet) and H6′ (3.16 ppm, 13.5 Hz, 3.0 Hz, doublet of doublets) share the 13.5 Hz ^2J and H6′ adds 13.5 Hz $^3J_{ax\text{-}ax}$; the H6′ 3.0 Hz coupling matches $^3J_{ax\text{-}eq}$, and the small unresolved H6 coupling matches $^3J_{eq\text{-}eq}$. H5 (3.06 ppm, 11.6 Hz doublet, unresolved) and H5′ (2.89 ppm, 12.0 Hz doublet of doublets, 3.5 Hz further doublet) share the 11.6 Hz ^2J to which H5′ adds a large splitting that matches $^3J_{ax\text{-}ax}$; the H5′ 3.5 Hz coupling matches $^3J_{ax\text{-}eq}$, and the unresolved H5 coupling matches $^3J_{eq\text{-}eq}$. The ^3J couplings within the ring indicate that 23b adopts a single chair, and the $^3J_{ax\text{-}eq}$ between H2 and H3 show that this is the **cis product**. The ester methylene protons have nearly identical chemical shifts, indicating that the C2-E1 bond rotates freely with almost no rotamer bias. The ethyl group protons show no evidence of exchange.

Table 1. Chemical shift assignments from 600 MHz

	23b		24b		
	^{1}H	^{13}C	^{1}H	^{13}C	Δδ
2	4.59	55.93	4.67	54.55	-1.4
3	3.95	62.94	3.96	57.14	-5.8
5	3.06	46.61	2.96	39.99	-6.6
5′	2.89		2.80		
6	3.74	41.05	3.51	41.58	+0.5
6′	3.16		3.25		
E1	2.37	31.00	3.18	36.03	+5.0
E1′			2.54		
E2		170.52		170.94	+0.4
Et1	3.62	60.49	4.07*	61.11	+0.6
Et2	0.96	13.95	1.19	14.20	+0.2

^{3}J analysis for 24b. H2 (4.67 ppm, 9.4 Hz, ~3Hz, doublet of doublets) shows a 9.4 Hz coupling to E1; with a pair of doublets of ~3 Hz, $^3J_{H2\text{-}H3}$ is a poor match to $^3J_{eq\text{-}eq}$ but it could be $^3J_{ax\text{-}eq}$ or result from averaging of $^3J_{eq\text{-}eq}$ with a little $^3J_{ax\text{-}ax}$. The broad H3 peak (3.95 ppm, 6 Hz FWHM, unresolved) could mask any doublet of about 3 Hz or smaller, and so is of little help. E1 (3.18 ppm, 16.3 Hz, 9.4 Hz, doublet of doublets) and E1′ (2.54 ppm, 16.3 Hz, 3.4 Hz, doublet of doublets) share a 16.3 Hz ^2J; the E1 9.4 Hz doublet is due to H2, as is the E1′ 3.4 Hz doublet. The different values for $^3J_{H2\text{-}E1}$ and $^3J_{H2\text{-}E1'}$ mean that any averaging about the C2-E1 bond must be highly rotamer biased, consistent with the E1/E1′ chemical shift differences. H5 (2.96 ppm, 11.9 Hz, 3.9 Hz, doublet of doublets) and H5′ (2.80 ppm, 12.2 Hz, 3.0 Hz, doublet of doublets) share the 12.2 Hz ^2J and H5 adds one coupling that is somewhat weaker than $^3J_{ax\text{-}ax}$ and another that is at the upper limit of or stronger than $^3J_{ax\text{-}eq}$. The two H5′ ~3.0 Hz couplings are too strong and too similar to correspond to both $^3J_{eq\text{-}eq}$ and $^3J_{ax\text{-}eq}$. H6 (3.51 ppm, 12.9 Hz, 3.0 Hz, doublet of doublets) and H6′ (3.25 ppm, 12.1, 3.5 Hz, doublet of doublets) share the 12.9 Hz ^2J and H6′ adds a strong coupling that seems weaker than $^3J_{ax\text{-}ax}$; the H6′ 3.5 Hz coupling could however be $^3J_{ax\text{-}eq}$. The lack of a small $^3J_{eq\text{-}eq}$ (< 2 Hz) between the H5s/H6s or between H2 and H3 indicates that 24b does not adopt a single chair; we infer that it averages between the diaxial (predominant) and diequatorial species. The multiplet pattern for the ethyl CH_2 is consistent with some kind of exchange.

References:

1. Reddy Guduru, S.K.; Chamakuri, S.; Raji, I.O.; MacKenzie, K.R.; Santini, C.; Young, D.W. Synthesis of Enantiomerically Pure 3-Substituted Piperazine-2-acetic Acid Esters as Intermediates for Library Production. *J. Org. Chem.* **2018**, *83*, 11777–11793.
2. Bisol, T.B.; Bortoluzzi, A.J.; Sa, M.M. Nucleophilic ring-opening of epoxide and aziridine acetates for the stereodivergent synthesis of β-hydroxy and β-amino γ-lactams. *J. Org. Chem.* **2011**, *76*, 948–962.

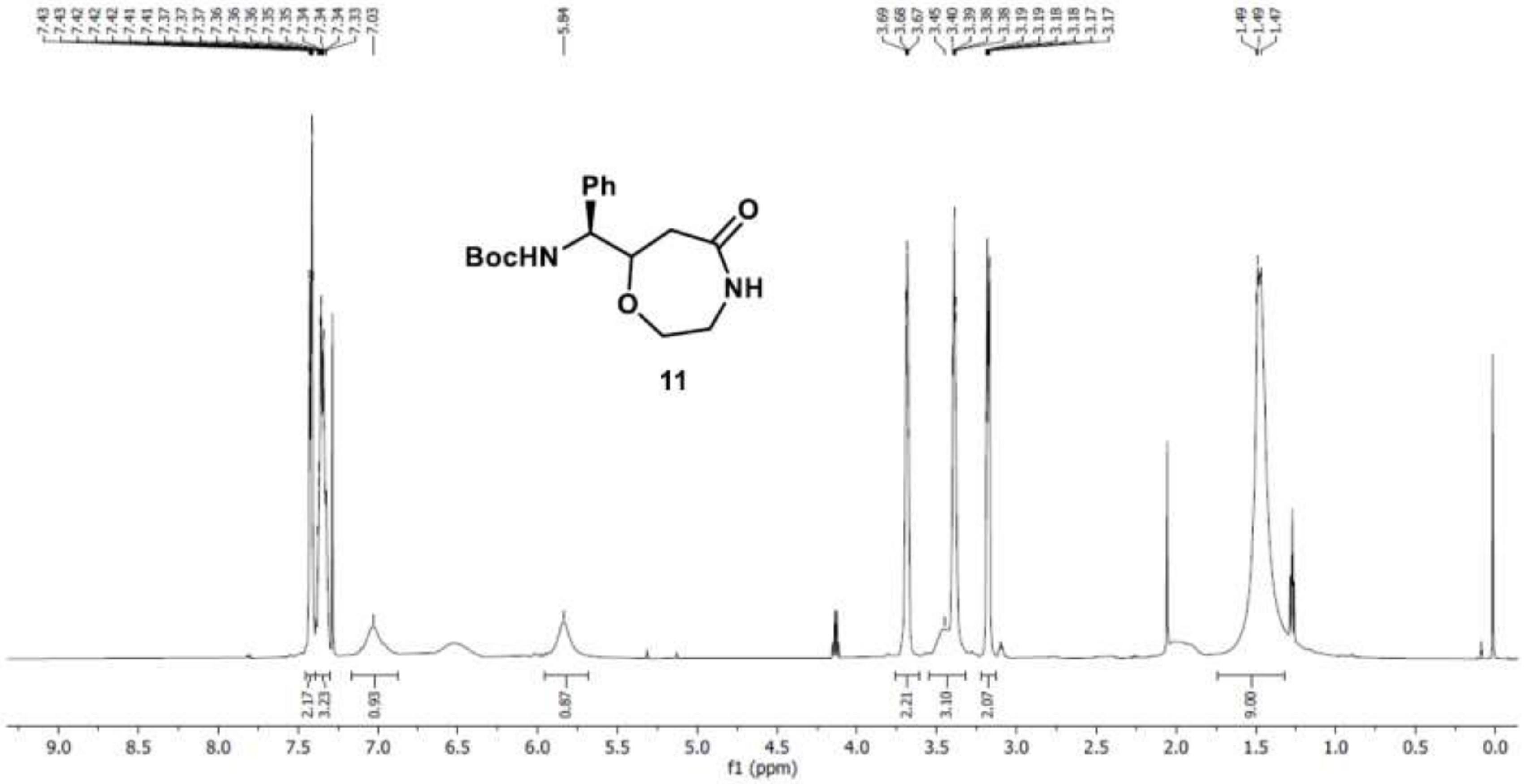
7.43
7.43
7.42
7.42
7.42
7.41
7.41
7.37
7.37
7.37
7.36
7.36
7.36
7.35
7.35
7.34
7.34
7.34
7.33
7.03
5.84
3.69
3.68
3.67
3.45
3.40
3.39
3.38
3.38
3.19
3.19
3.18
3.18
3.17
3.17
1.49
1.49
1.47
Ph
O
BocHN
NH
11
2.17
3.23
0.93
0.87
2.21
3.10
2.07
9.00
9.0
8.5
8.0
7.5
7.0
6.5
6.0
5.5
5.0
4.5
4.0
3.5
3.0
2.5
2.0
1.5
1.0
0.5
0.0
f1 (ppm)

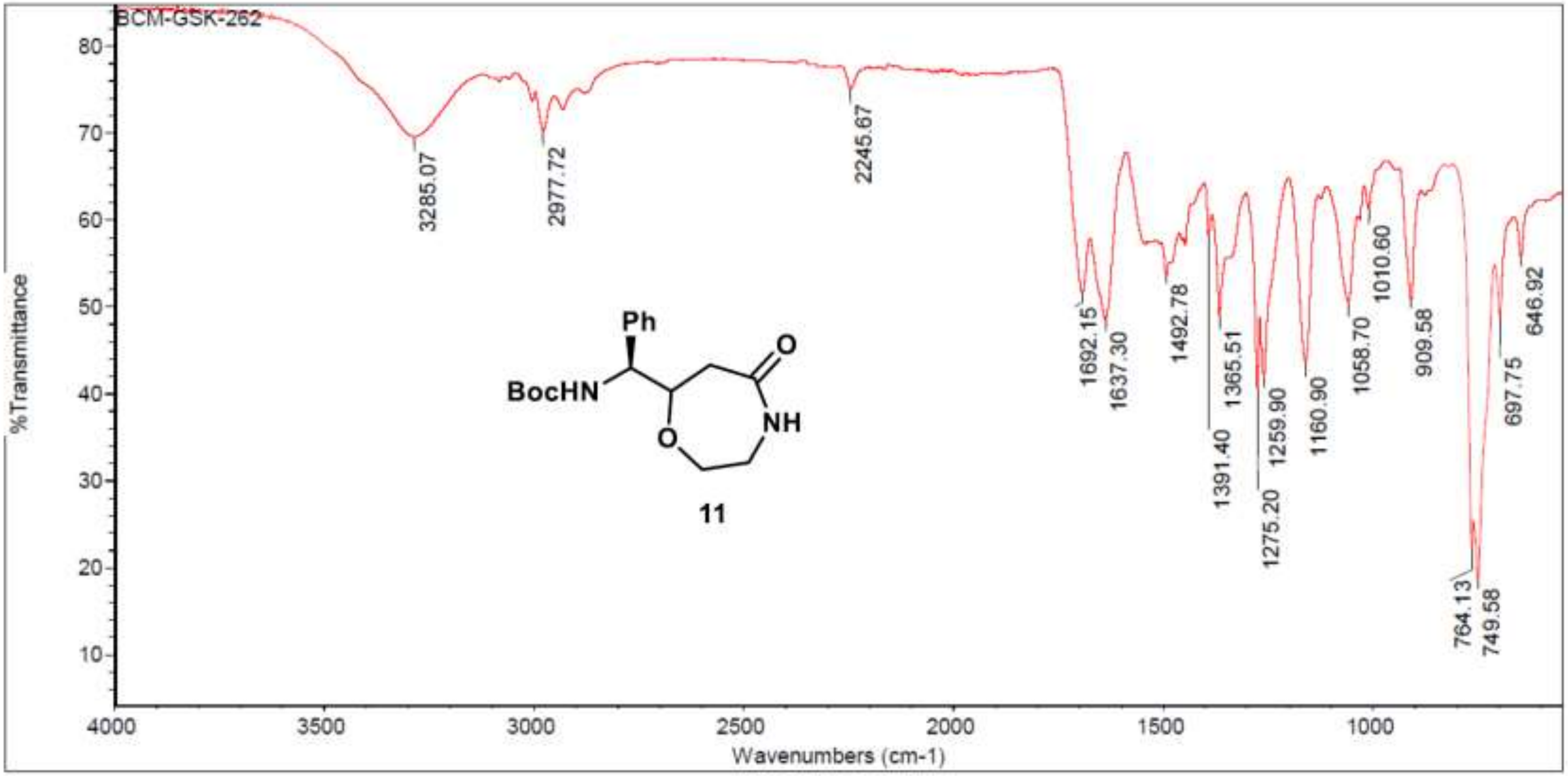
BCM-GSK-262
%Transmittance
Wavenumbers (cm-1)
3285.07
2977.72
2245.67
1692.15
1637.30
1492.78
1391.40
1365.51
1275.20
1259.90
1160.90
1058.70
1010.60
909.58
764.13
749.58
697.75
646.92
Ph
BocHN
O
NH
11

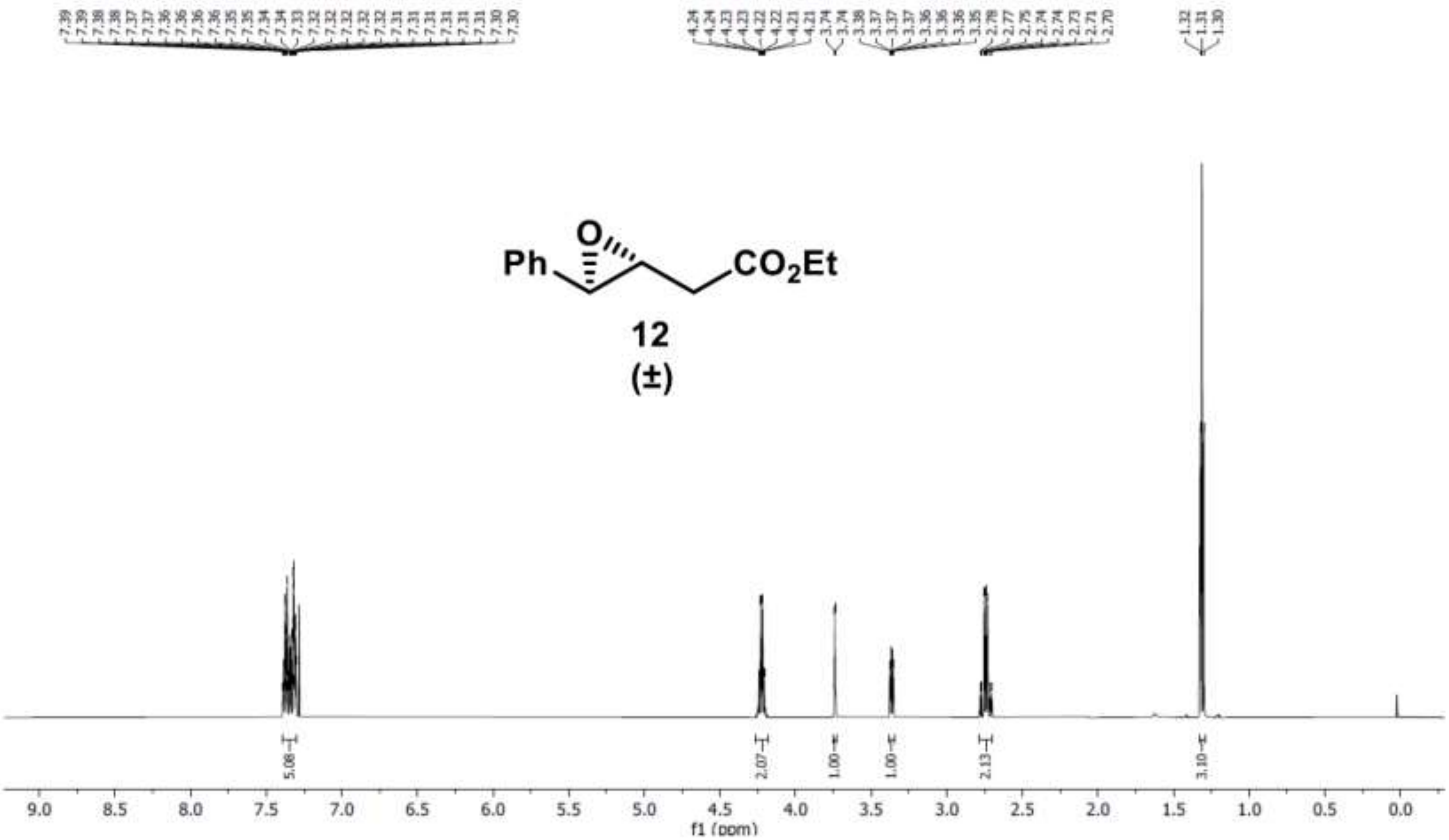

7.39
7.39
7.38
7.38
7.37
7.37
7.36
7.36
7.36
7.36
7.35
7.35
7.34
7.34
7.33
7.32
7.32
7.32
7.32
7.32
7.31
7.31
7.31
7.31
7.31
7.30
7.30
4.24
4.24
4.23
4.23
4.22
4.22
4.21
4.21
3.74
3.74
3.38
3.37
3.37
3.37
3.36
3.36
3.36
3.35
2.78
2.77
2.75
2.74
2.74
2.73
2.71
2.70
1.32
1.31
1.30
Ph
O
CO_2Et
5.08
2.07
1.00
1.00
2.13
3.10
9.0
8.5
8.0
7.5
7.0
6.5
6.0
5.5
5.0
4.5
4.0
3.5
3.0
2.5
2.0
1.5
1.0
0.5
0.0
f1 (ppm)

12
(±)

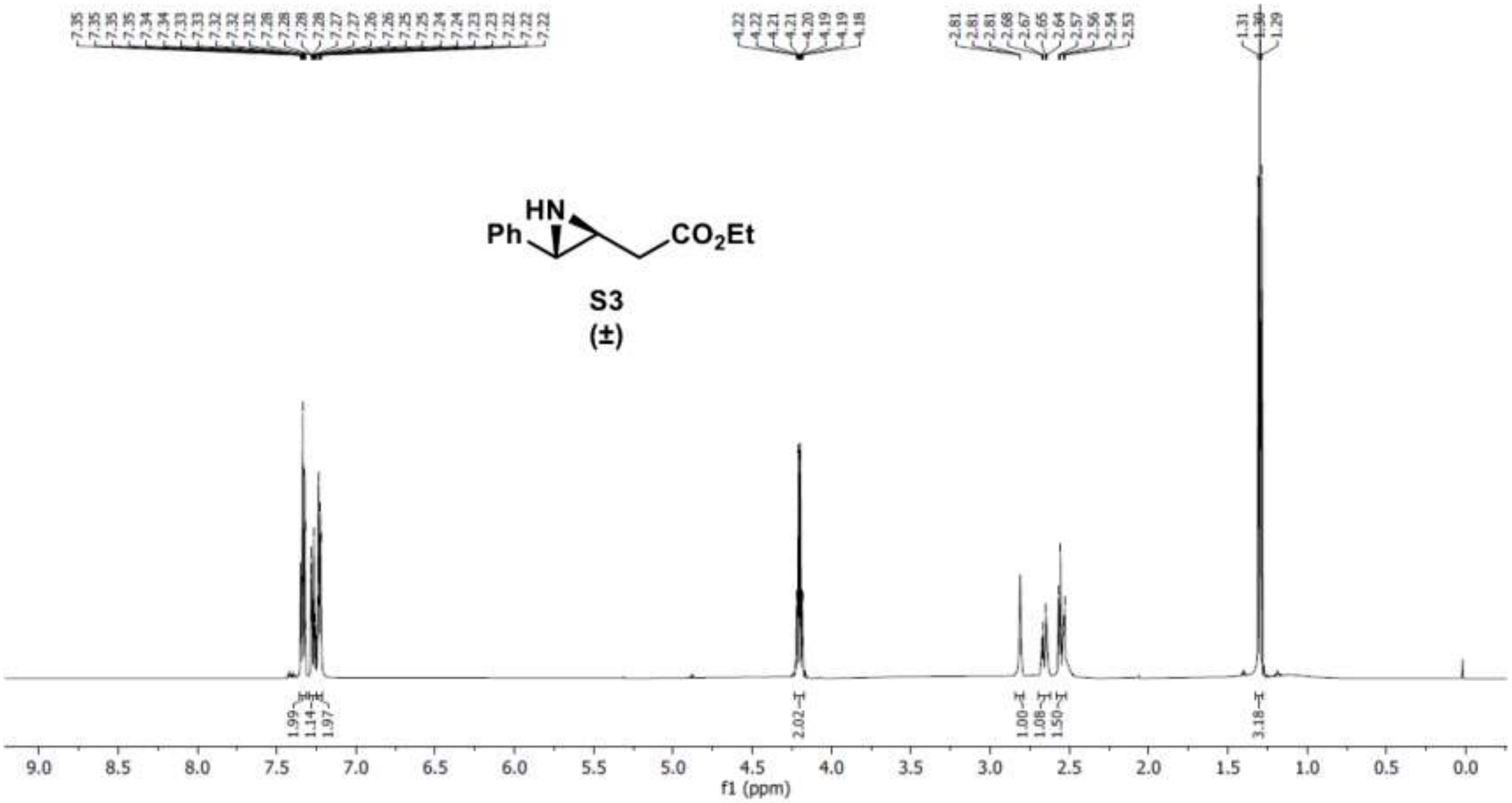
HN
Ph
CO2Et
S3
(±)
f1 (ppm)

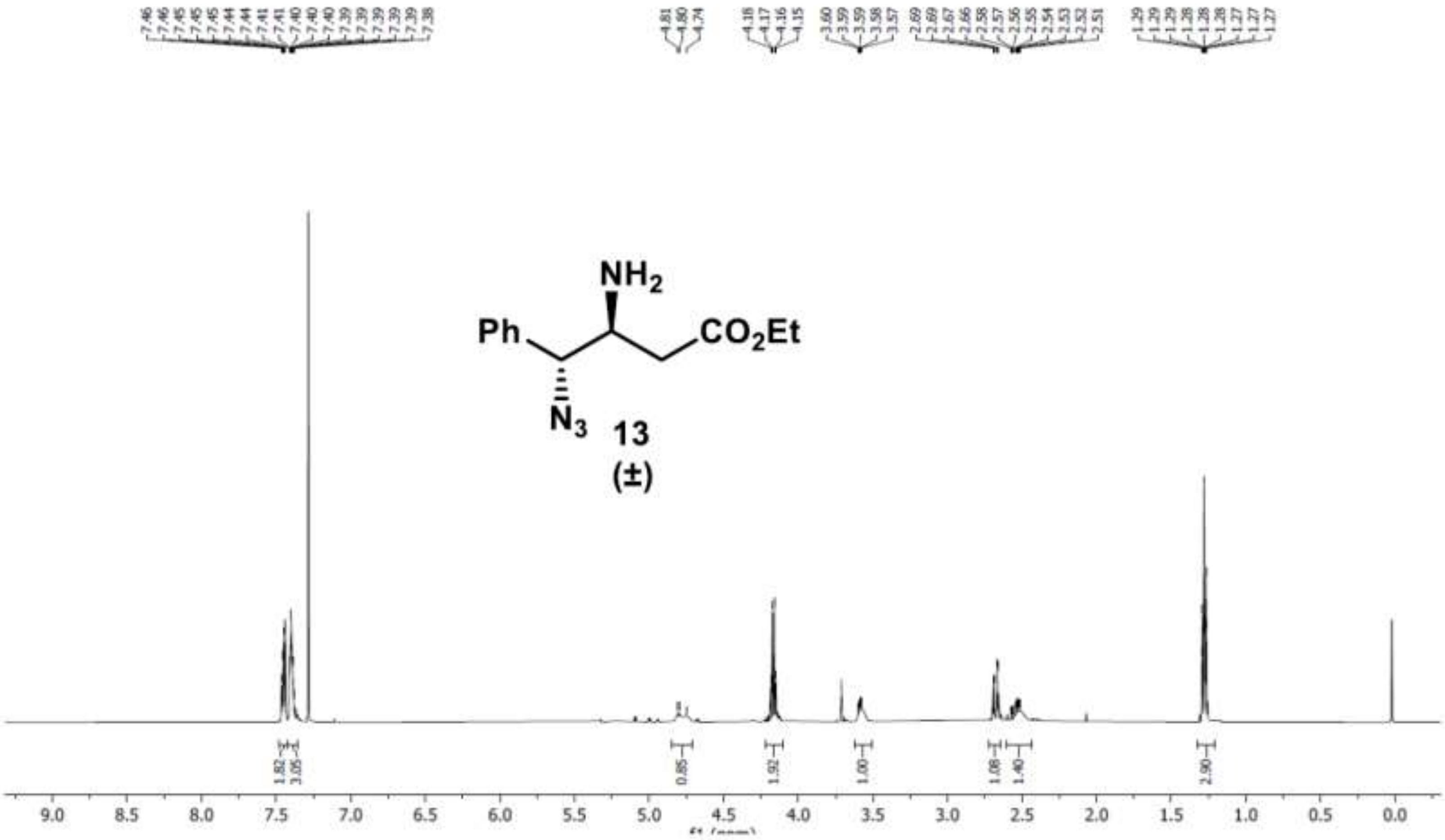

7.46 7.46 7.45 7.45 7.45 7.44 7.44 7.41 7.41 7.40 7.40 7.40 7.39 7.39 7.39 7.39 7.39 7.38
4.81 4.80 4.74
4.18 4.17 4.16 4.15
3.60 3.59 3.59 3.58 3.57
2.69 2.69 2.67 2.66 2.58 2.57 2.56 2.55 2.54 2.53 2.52 2.51
1.29 1.29 1.29 1.28 1.28 1.28 1.27 1.27 1.27
NH2
Ph
CO2Et
N3
13
(±)
1.82
3.05
0.85
1.92
1.00
1.08
1.40
2.90
9.0 8.5 8.0 7.5 7.0 6.5 6.0 5.5 5.0 4.5 4.0 3.5 3.0 2.5 2.0 1.5 1.0 0.5 0.0

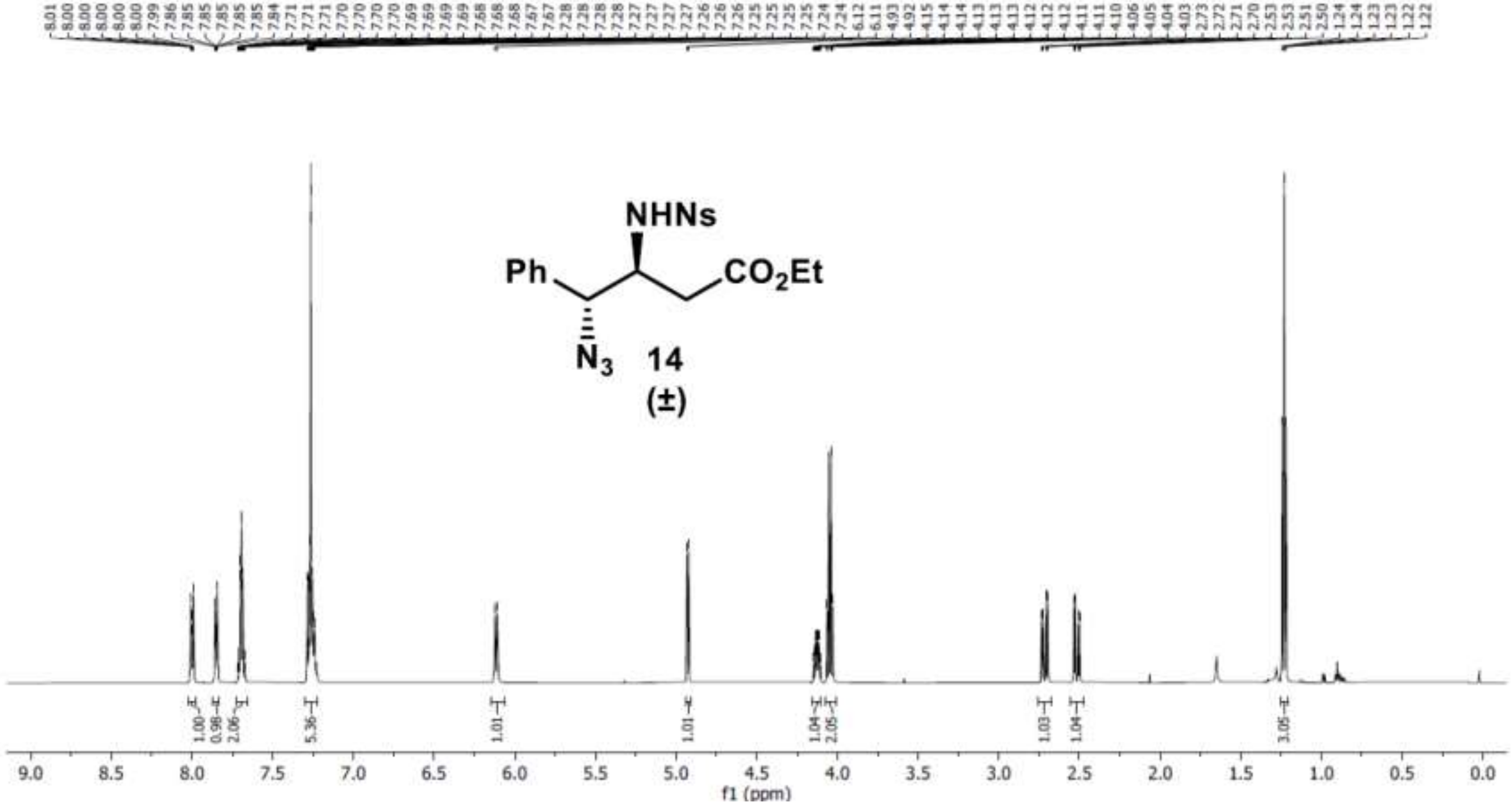
NHNs
Ph
CO_2Et
N_3
14
(±)
f1 (ppm)

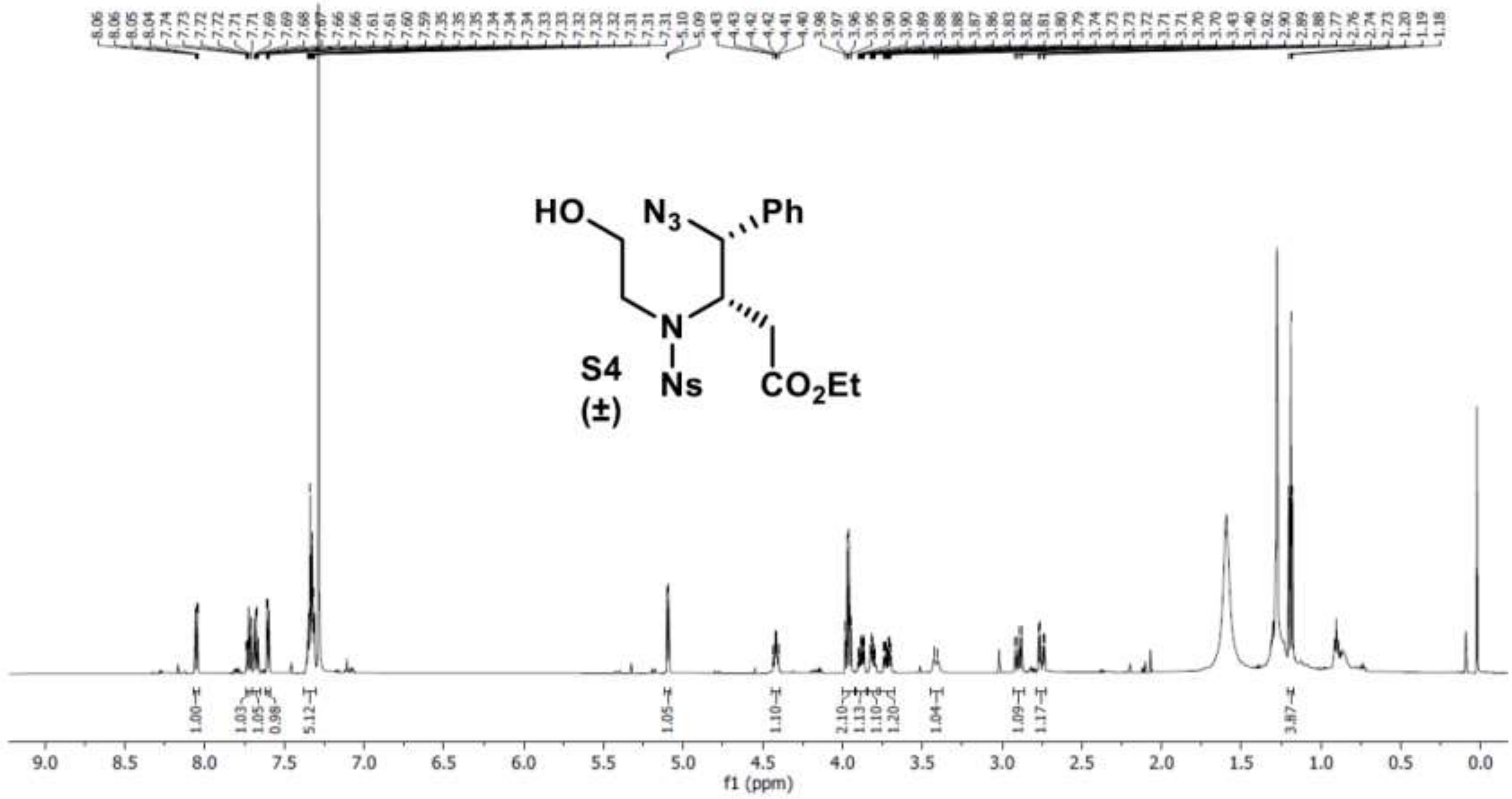

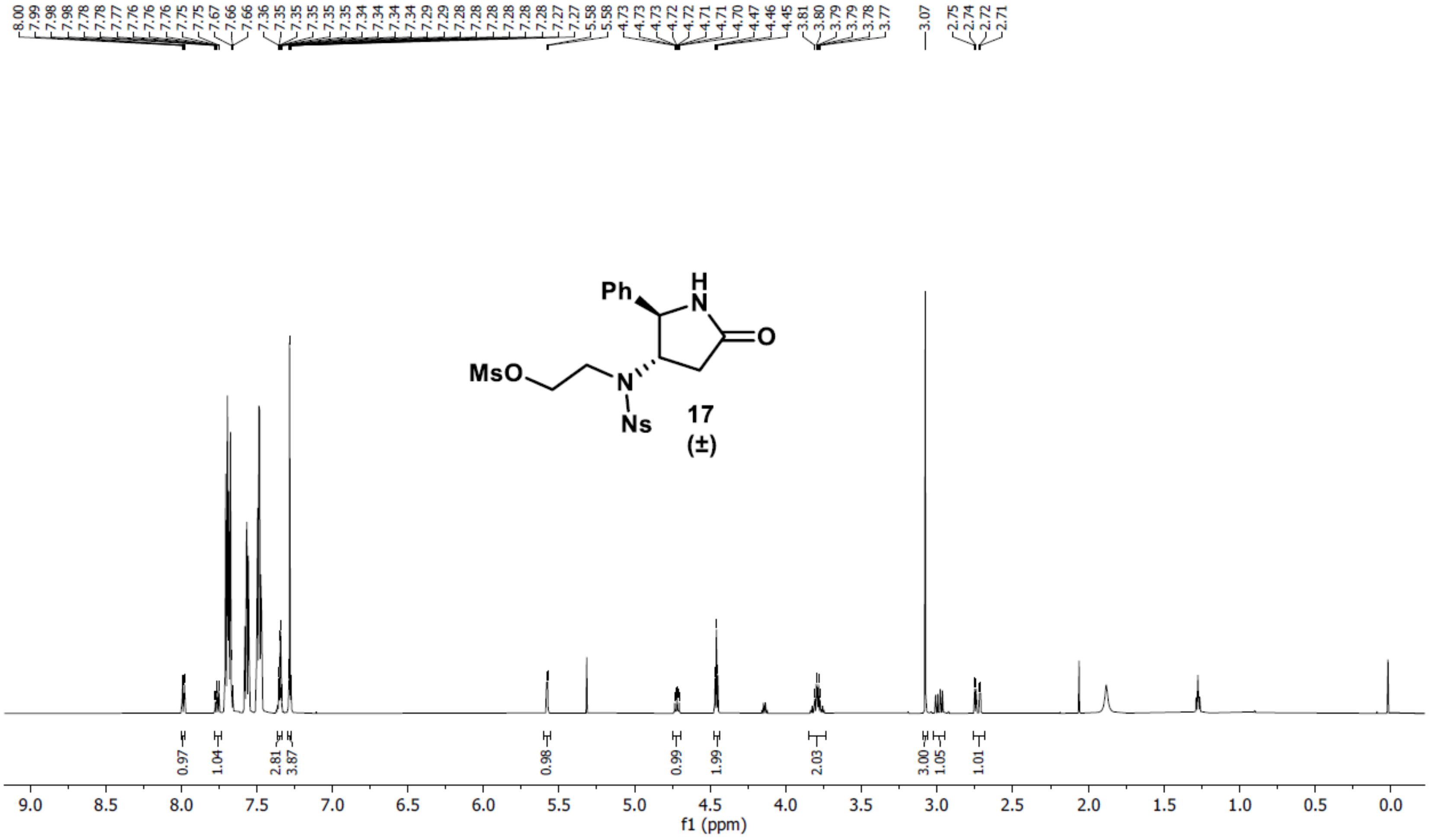

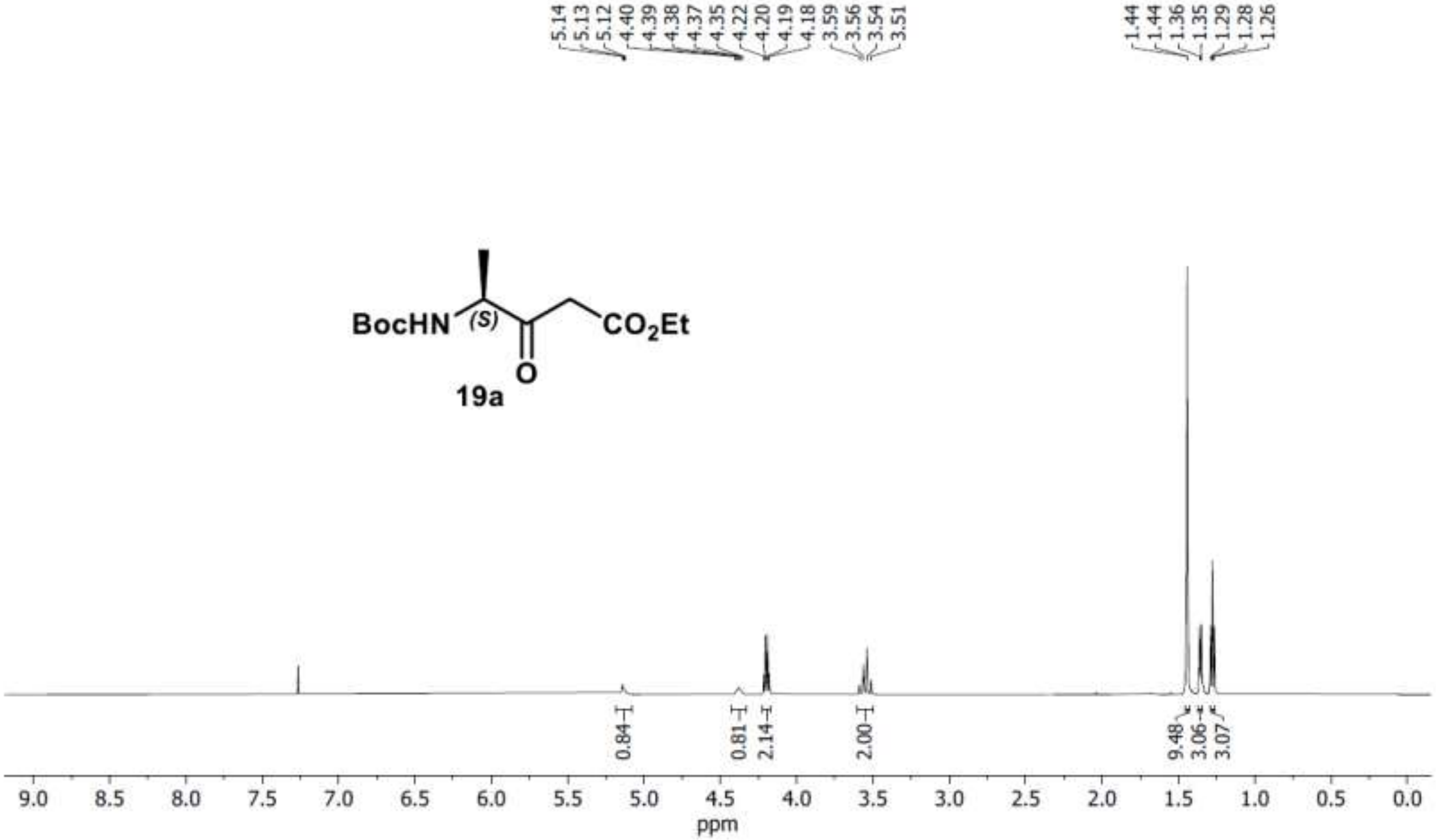
5.14
5.13
5.12
4.40
4.39
4.38
4.37
4.35
4.22
4.20
4.19
4.18
3.59
3.56
3.54
3.51
1.44
1.44
1.36
1.35
1.29
1.28
1.26
BocHN
(S)
O
CO2Et
19a
0.84
0.81
2.14
2.00
9.48
3.06
3.07
9.0
8.5
8.0
7.5
7.0
6.5
6.0
5.5
5.0
4.5
4.0
3.5
3.0
2.5
2.0
1.5
1.0
0.5
0.0
ppm

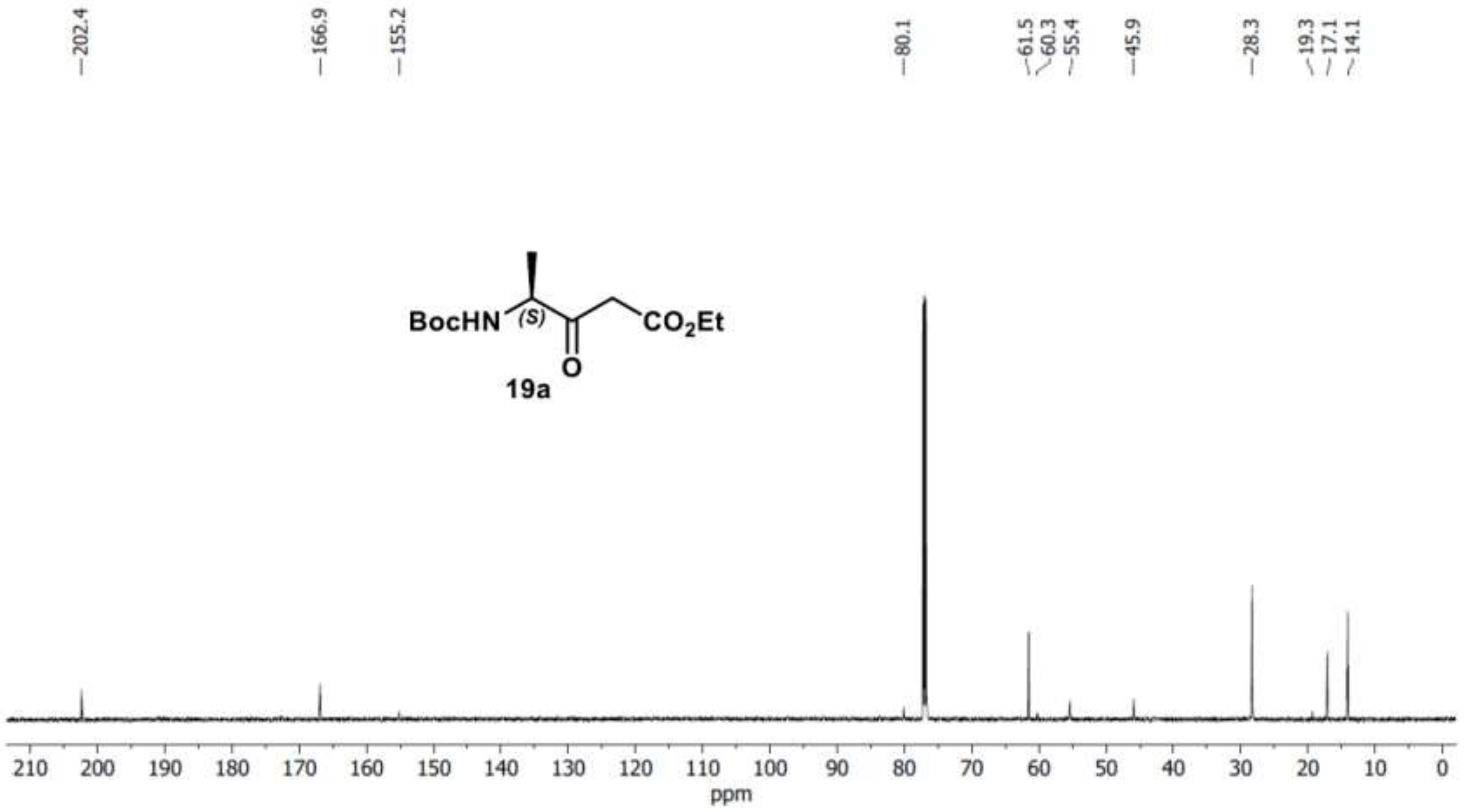
202.4
166.9
155.2
80.1
61.5
60.3
55.4
45.9
28.3
19.3
17.1
14.1
BocHN
(S)
O
CO2Et
19a
210 200 190 180 170 160 150 140 130 120 110 100 90 80 70 60 50 40 30 20 10 0
ppm

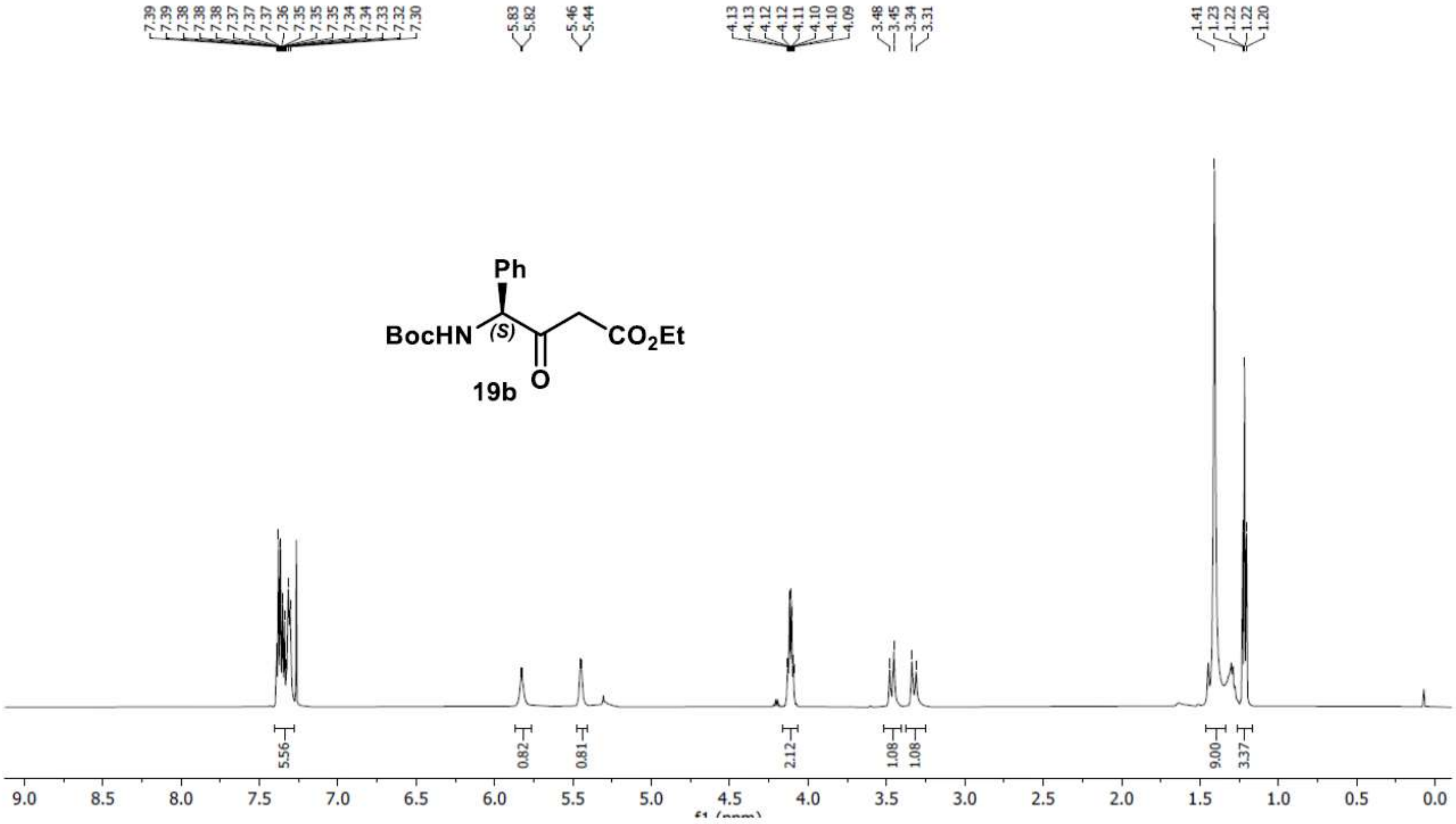
7.39 7.39 7.38 7.38 7.37 7.37 7.37 7.36 7.35 7.35 7.35 7.34 7.34 7.33 7.32 7.30
5.83 5.82
5.46 5.44
4.13 4.13 4.12 4.12 4.11 4.10 4.10 4.09
3.48 3.45 3.34 3.31
1.41 1.23 1.22 1.22 1.20
Ph
BocHN
(S)
CO2Et
O
19b
5.56
0.82
0.81
2.12
1.08
1.08
9.00
3.37
9.0 8.5 8.0 7.5 7.0 6.5 6.0 5.5 5.0 4.5 4.0 3.5 3.0 2.5 2.0 1.5 1.0 0.5 0.0
f1 (ppm)

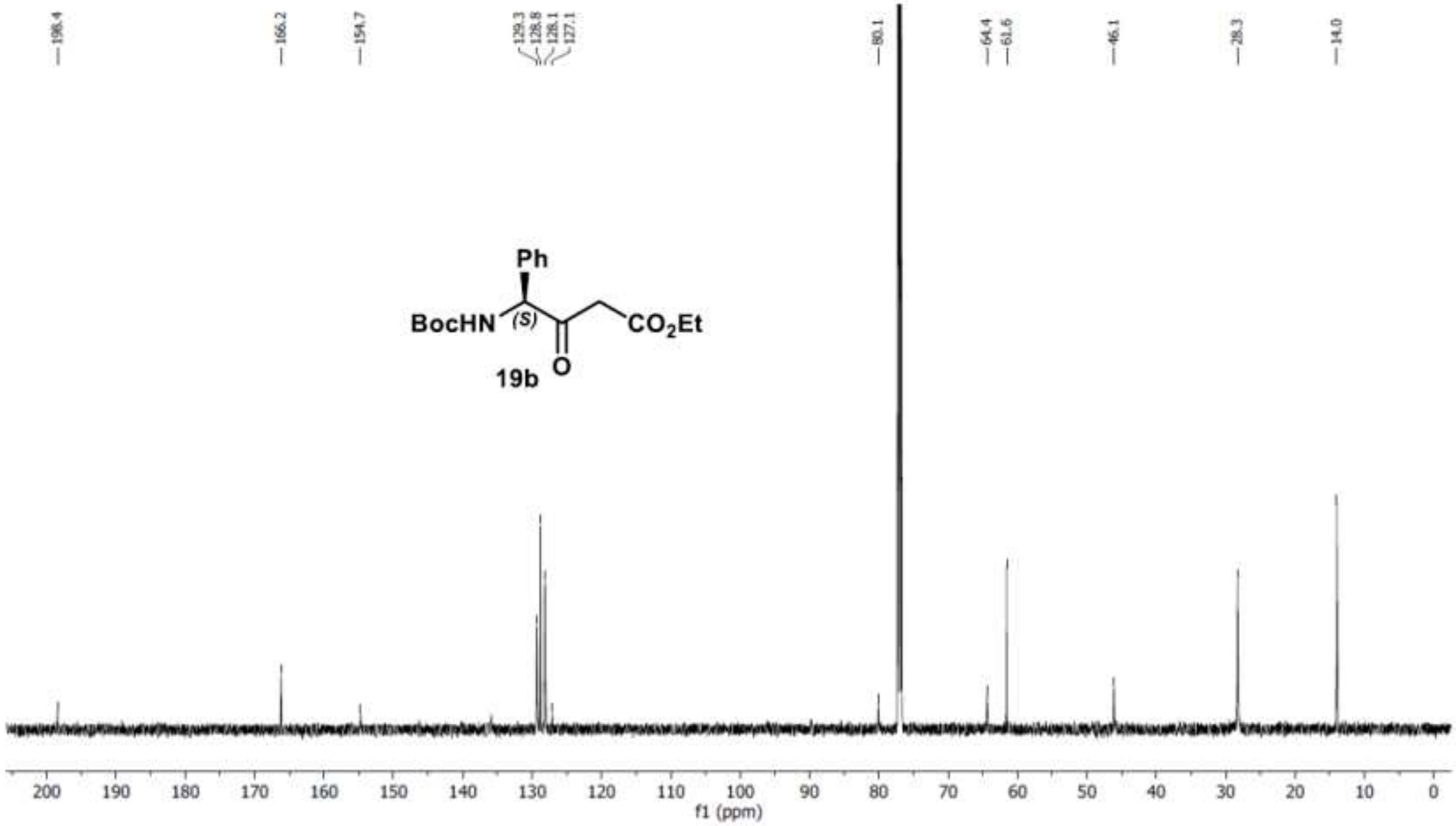
198.4
166.2
154.7
129.3
128.8
128.1
127.1
80.1
64.4
61.6
46.1
28.3
14.0
Ph
BocHN
(S)
CO2Et
O
19b
200 190 180 170 160 150 140 130 120 110 100 90 80 70 60 50 40 30 20 10 0
f1 (ppm)

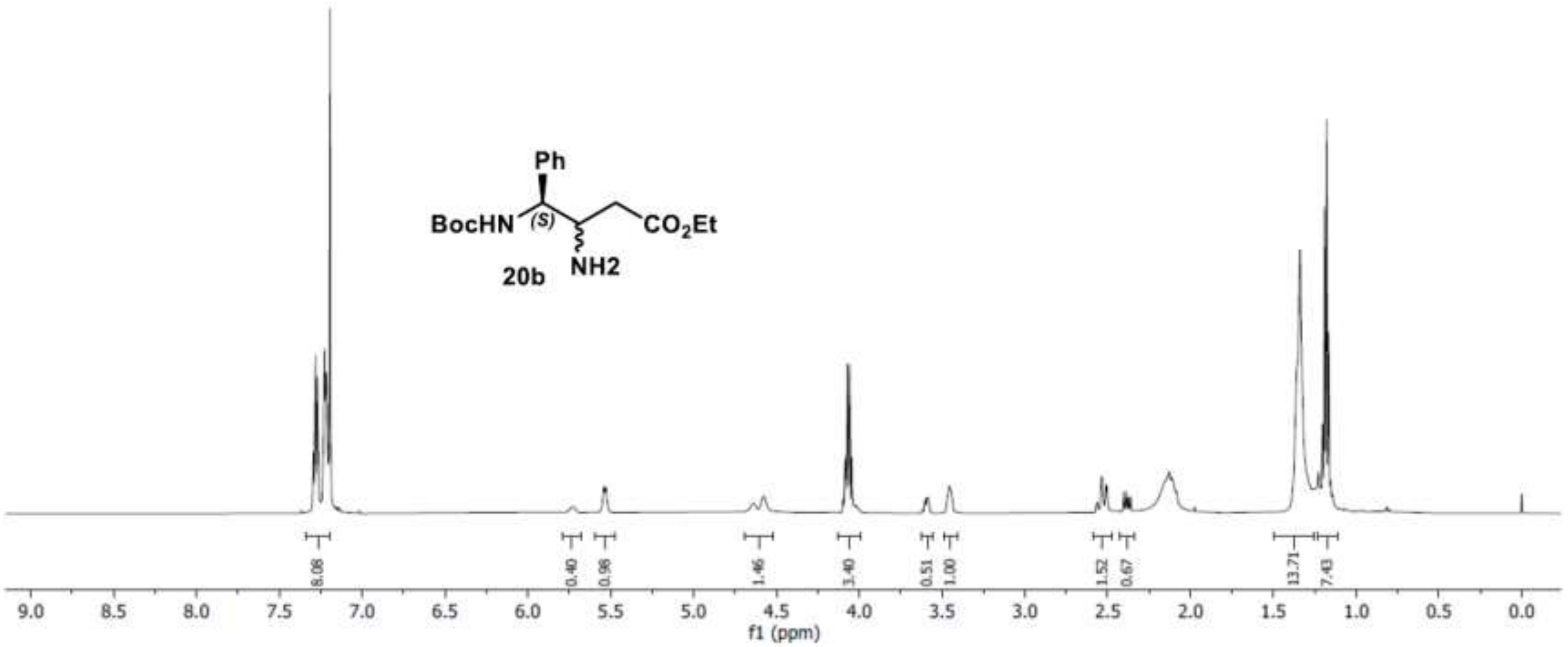

Ph
BocHN
(S)
CO2Et
NH2
20b
8.08
0.40
0.98
1.46
3.40
0.51
1.00
1.52
0.67
13.71
7.43
9.0
8.5
8.0
7.5
7.0
6.5
6.0
5.5
5.0
4.5
4.0
3.5
3.0
2.5
2.0
1.5
1.0
0.5
0.0
f1 (ppm)

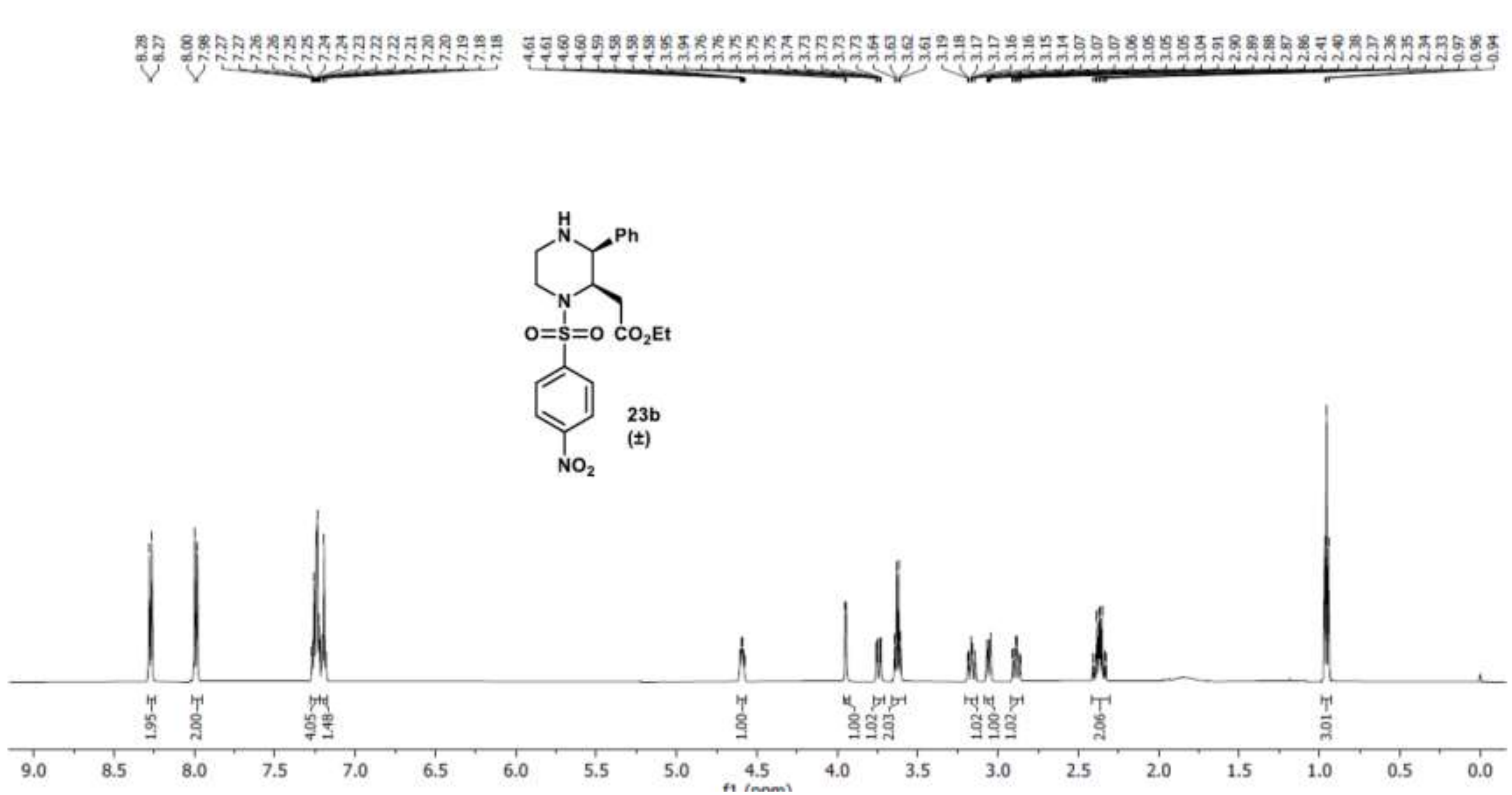
H
N
Ph
N
O=S=O
CO2Et
23b
(±)
NO2
f1 (ppm)

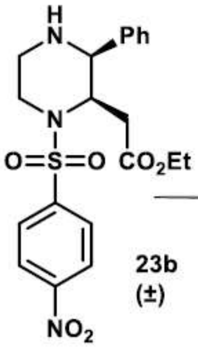

H
N
Ph
N
O=S=O
CO2Et
NO2

23b
(±)

COSY

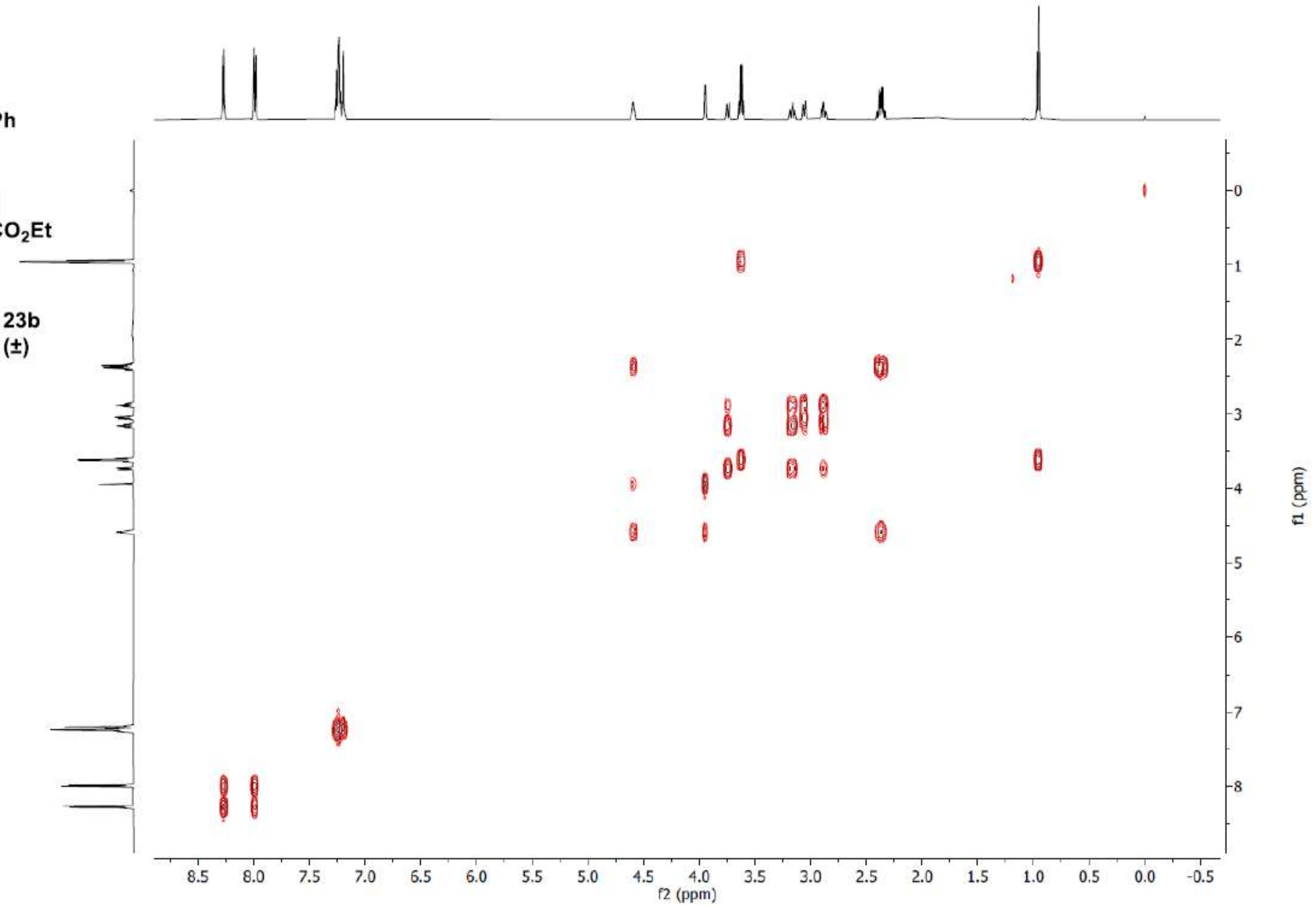

8.5 8.0 7.5 7.0 6.5 6.0 5.5 5.0 4.5 4.0 3.5 3.0 2.5 2.0 1.5 1.0 0.5 0.0 -0.5
f2 (ppm)
0 1 2 3 4 5 6 7 8
f1 (ppm)

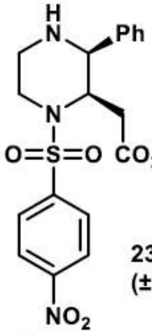

23b
(±)

HMBC

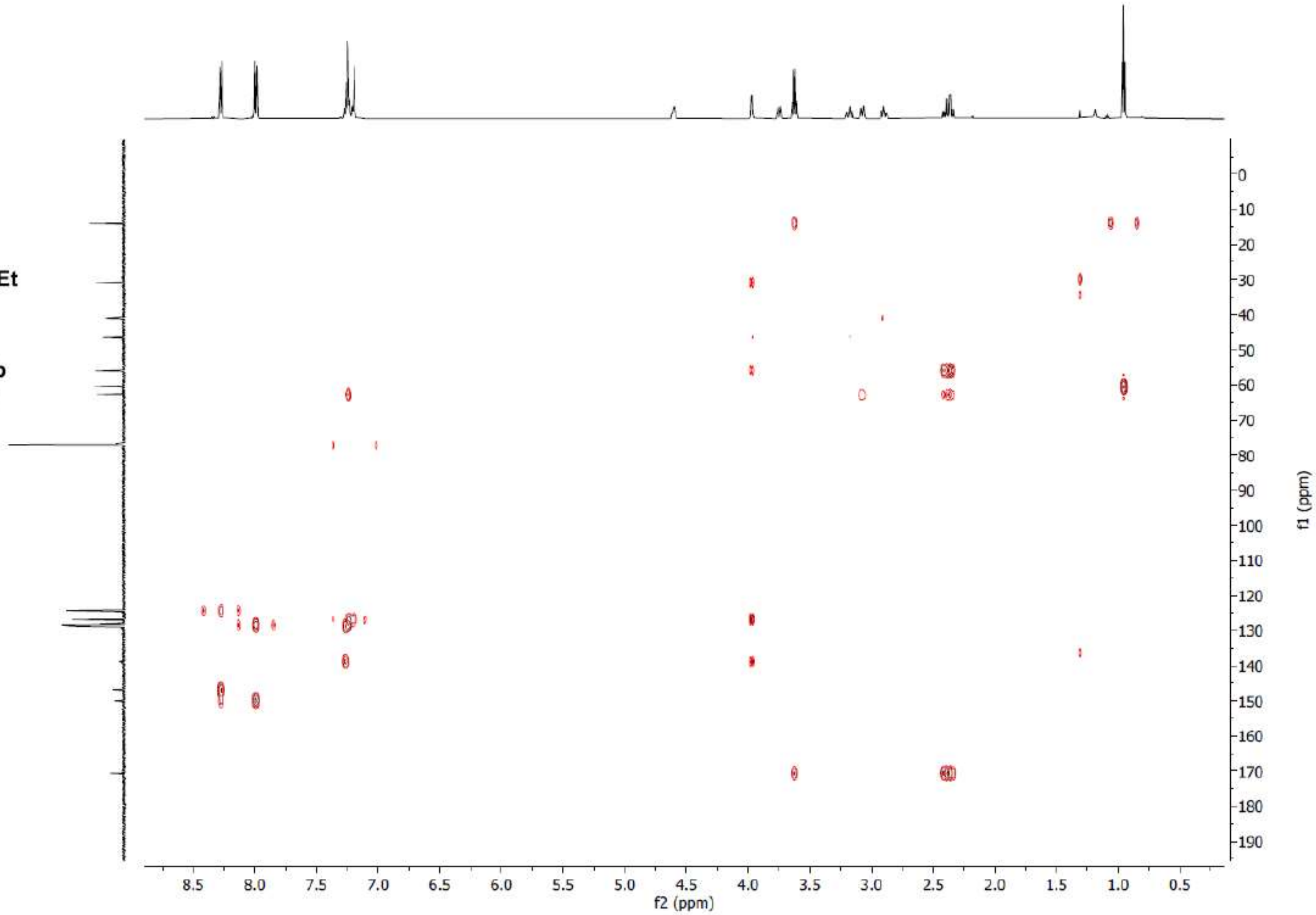

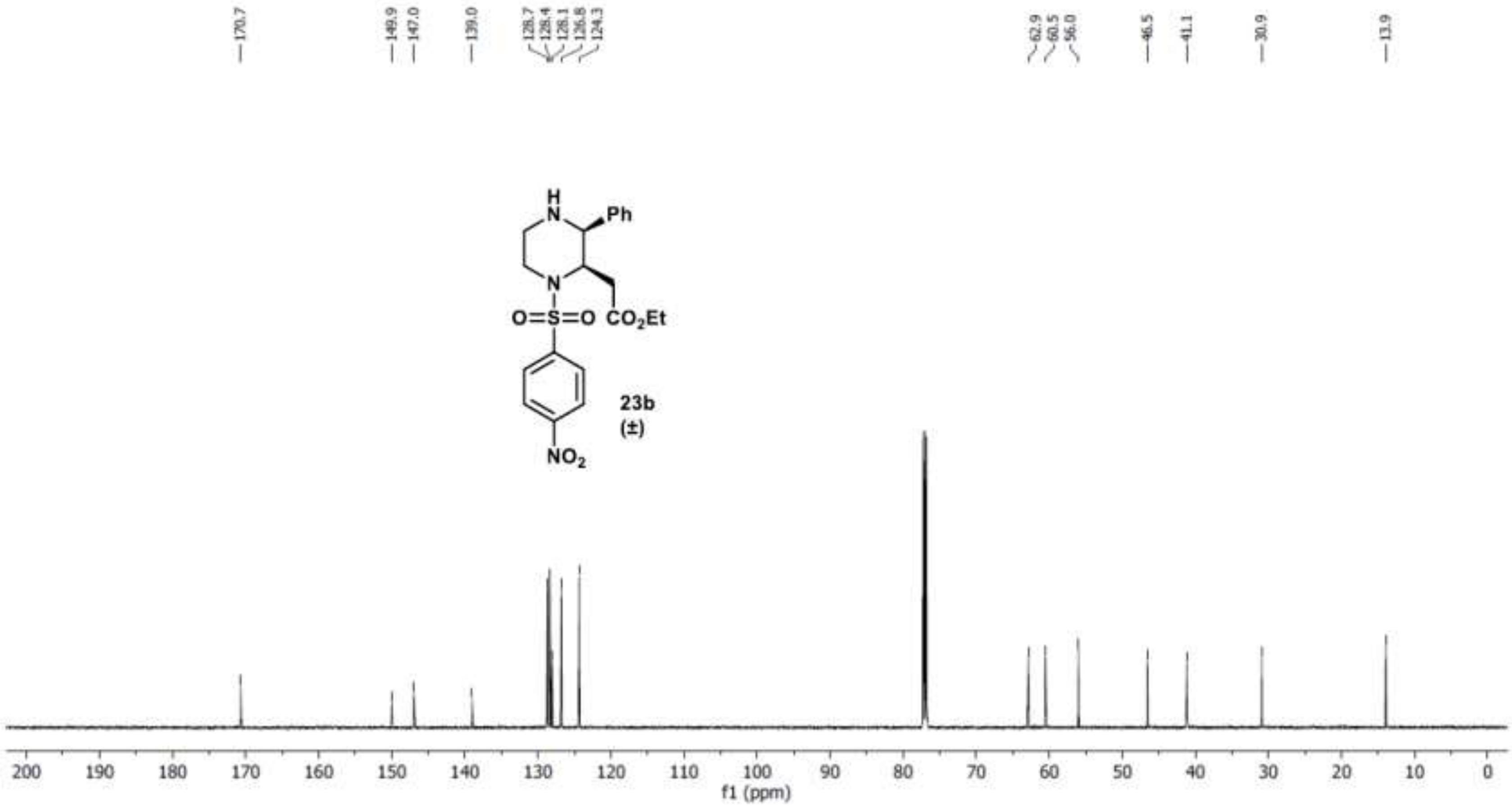
170.7
149.9
147.0
139.0
128.7
128.4
128.1
126.8
124.3
62.9
60.5
56.0
46.5
41.1
30.9
13.9
Ph
CO_2Et
NO_2
23b
(±)
f1 (ppm)

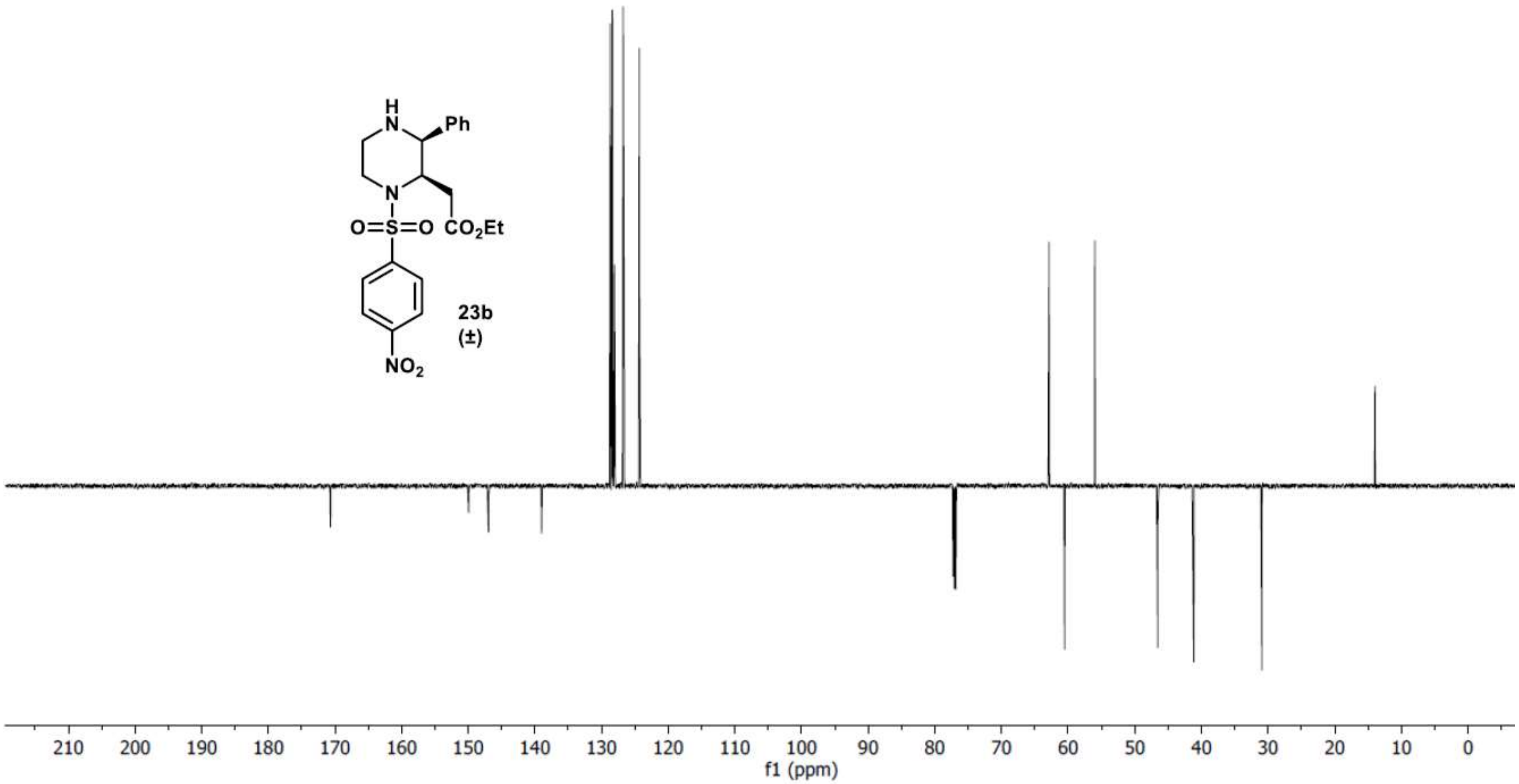
H
N
Ph
N
O=S=O
CO2Et
NO2
23b
(±)
210 200 190 180 170 160 150 140 130 120 110 100 90 80 70 60 50 40 30 20 10 0
f1 (ppm)

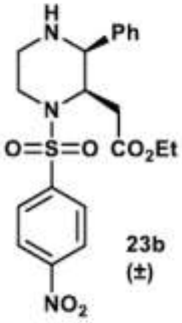

23b
(±)

HSQC

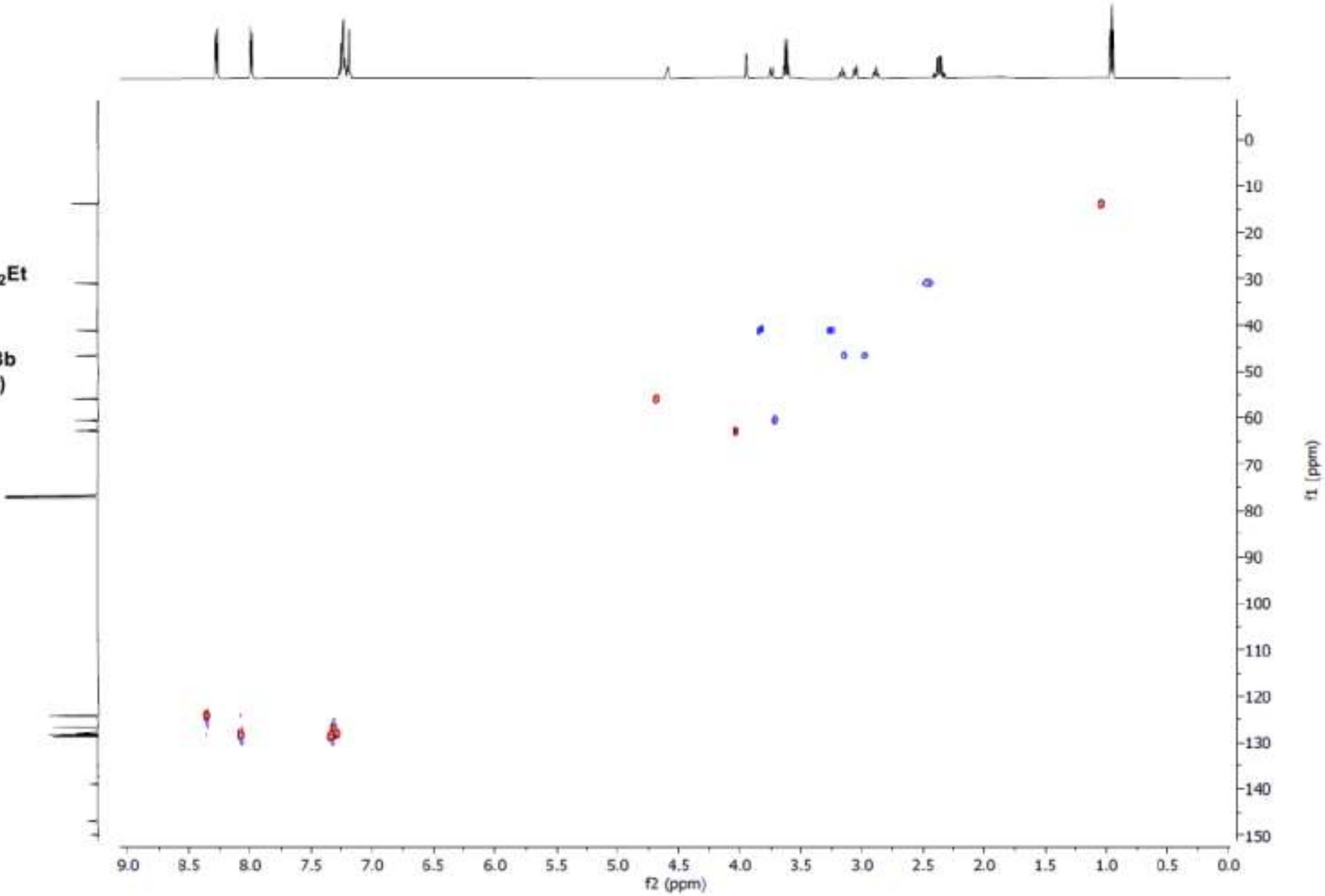

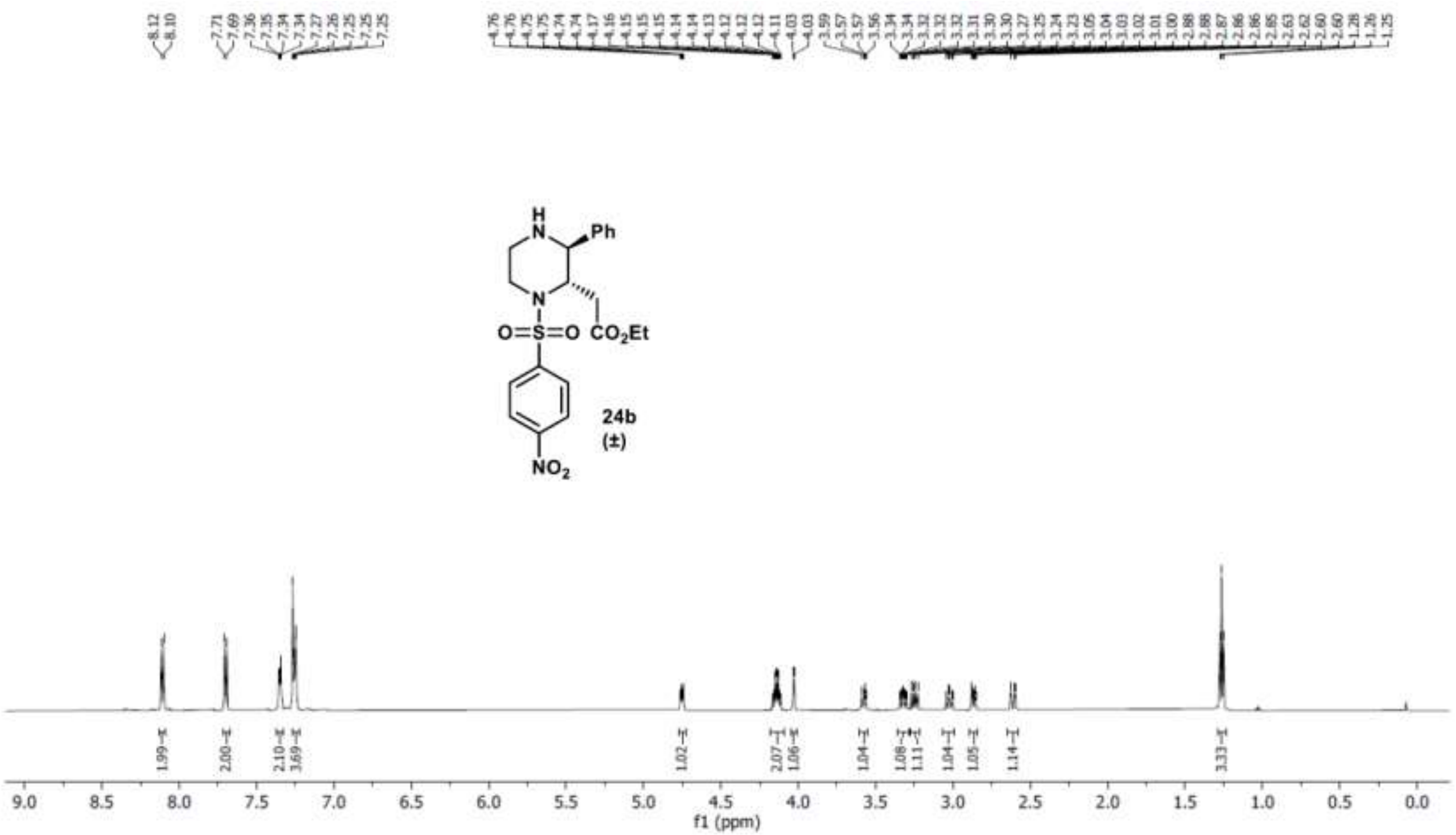

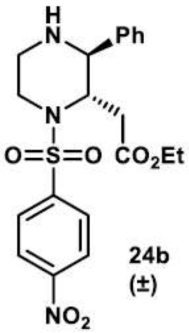

24b
(±)

COSY

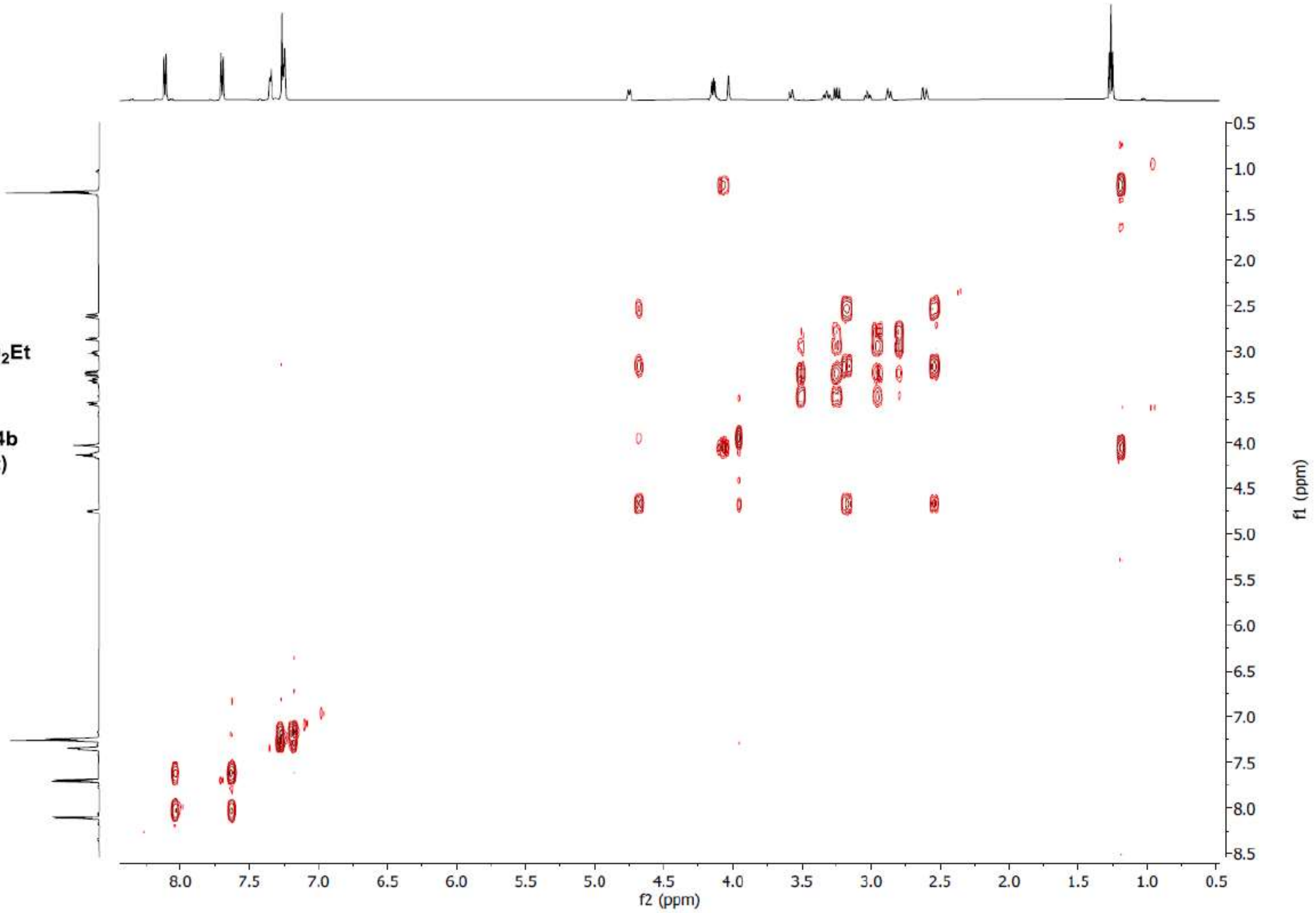

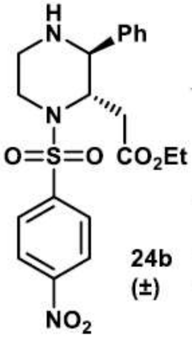

24b
(±)

HMBC

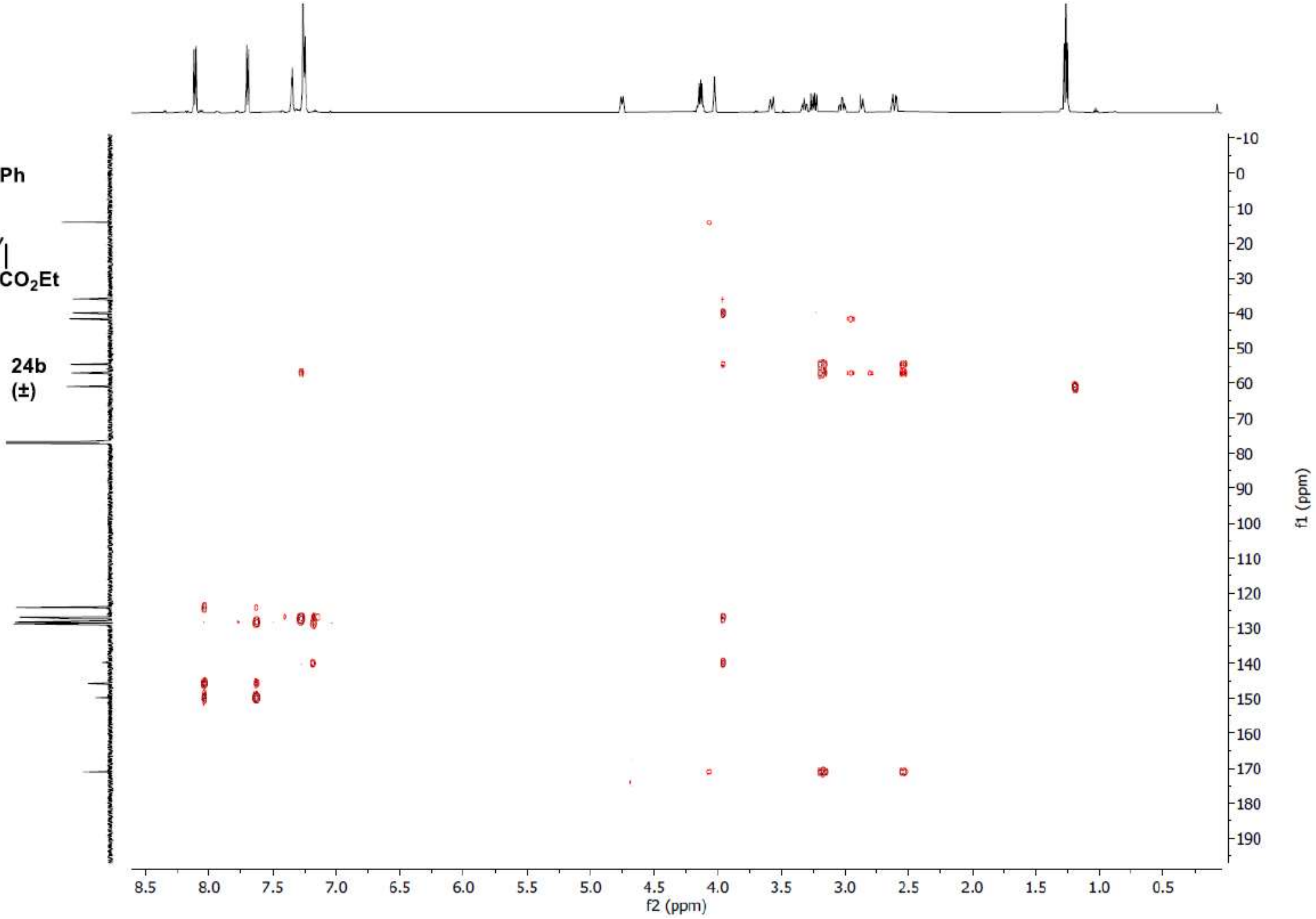

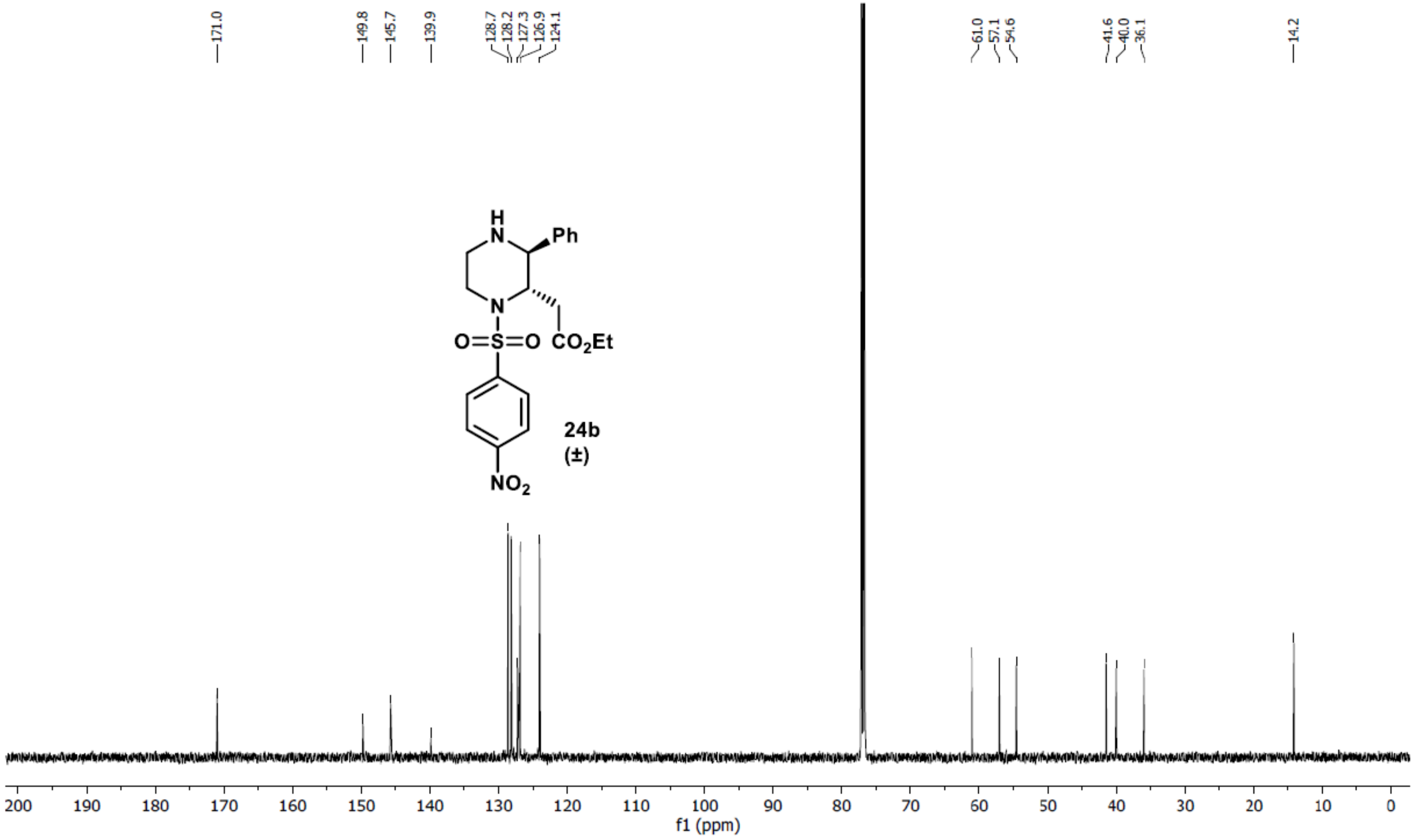
171.0
149.8
145.7
139.9
128.7
128.2
127.3
126.9
124.1
61.0
57.1
54.6
41.6
40.0
36.1
14.2
Ph
O=S=O
CO2Et
NO2
24b
(±)
200 190 180 170 160 150 140 130 120 110 100 90 80 70 60 50 40 30 20 10 0
f1 (ppm)

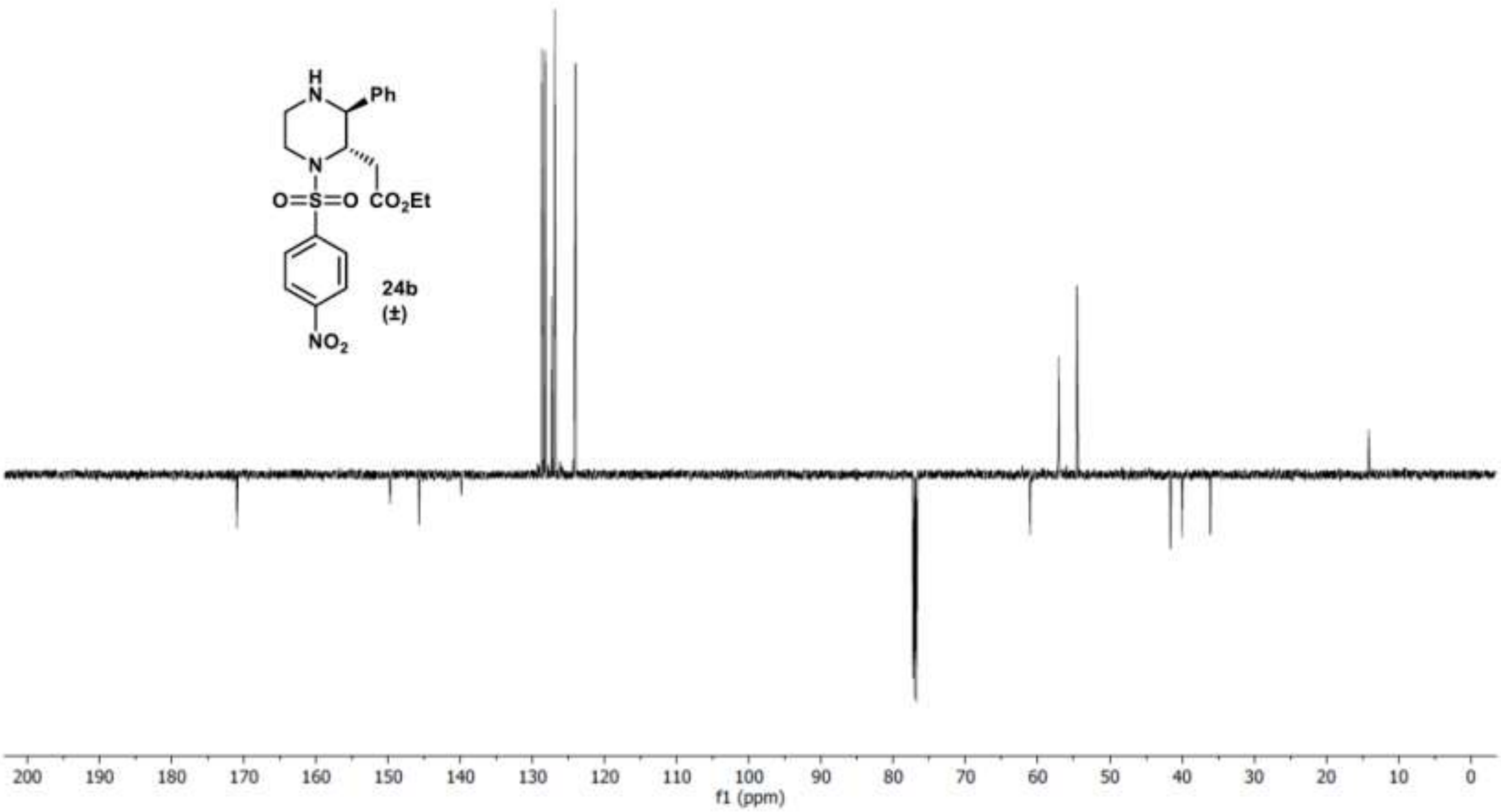

H
N
Ph
O=S=O
CO2Et
NO2
24b
(±)
200 190 180 170 160 150 140 130 120 110 100 90 80 70 60 50 40 30 20 10 0
f1 (ppm)

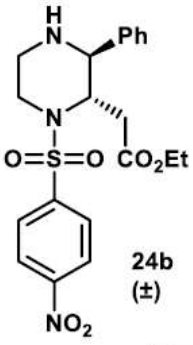

24b
(±)

HSQC

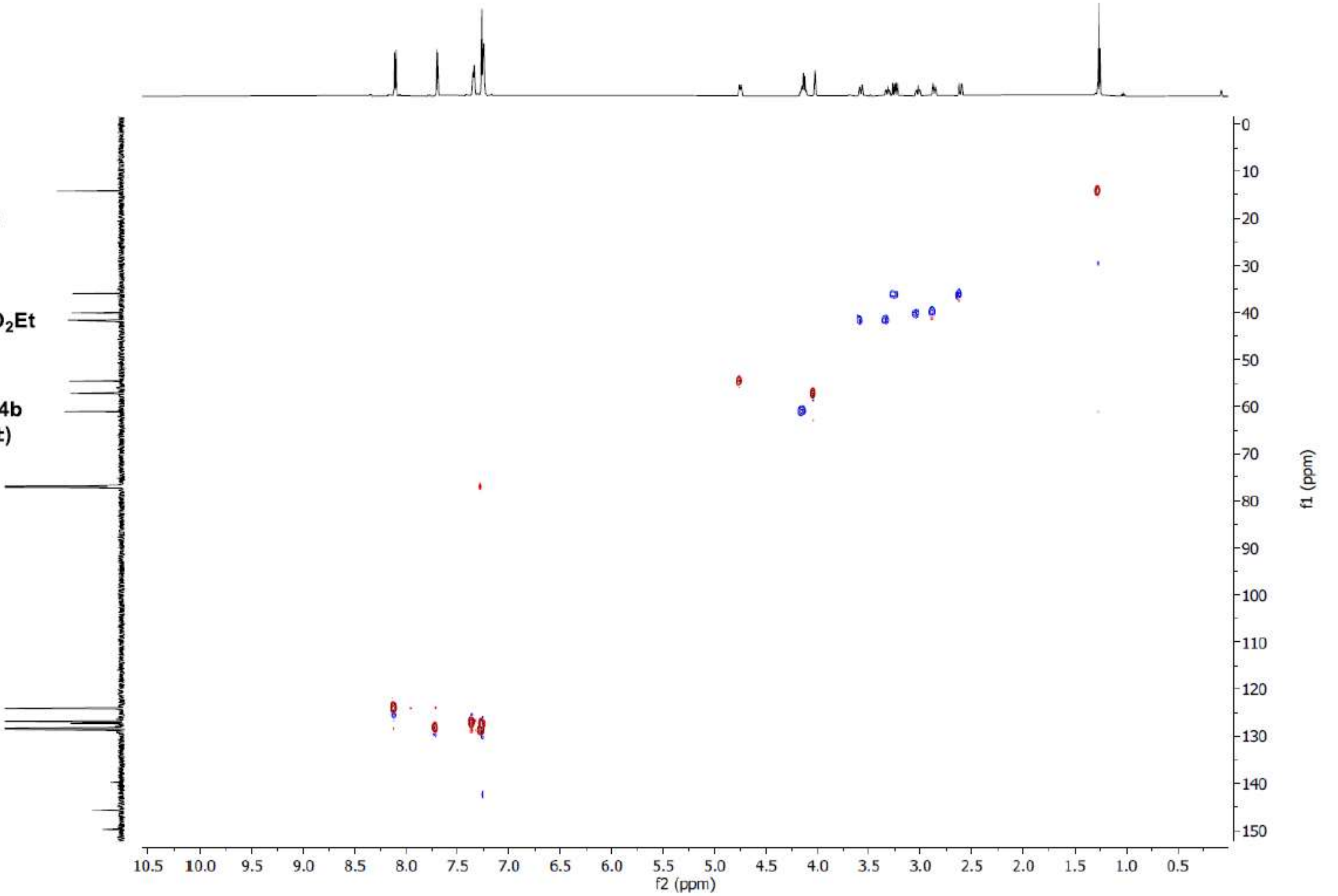

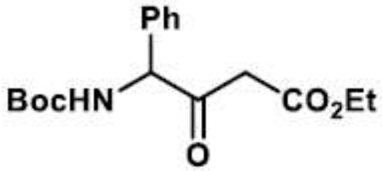

Data File C:\Chem32\1\Data\Srinivas\IT-4713 Racemic 2022-02-01 12-24-29.D
Sample Name: IT-4713 Racemic

```
=====================================================================
Acq. Operator   : SYSTEM
Sample Operator : SYSTEM
Acq. Instrument : CHIRAL                        Location :   2
Injection Date  : 2/1/2022 12:31:15 PM
                                             Inj Volume : 5.000 µl
Acq. Method     : C:\CHEM32\1\METHODS\IA_nHep_Ethanol_Custom.M
Last changed    : 2/1/2022 12:30:24 PM by SYSTEM
Analysis Method : C:\Chem32\1\Methods\Method_develop_Chiral_IF_nHep_Ethanol.M
Last changed    : 3/18/2021 4:41:28 PM by SYSTEM
Method Info     : method development for normal phase chiral separations ChiralPak IF with n-
                  Heptane and Ethanol

Additional Info : Peak(s) manually integrated
```

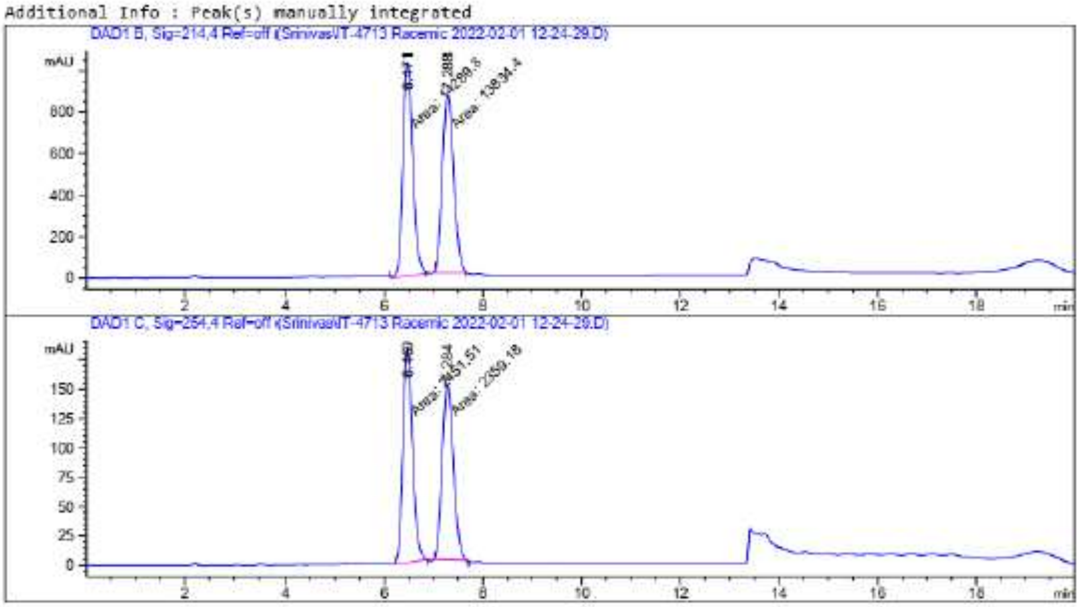


```
=====================================================================
                         Area Percent Report
=====================================================================

Sorted By             :      Signal
Multiplier            :      1.0000
Dilution              :      1.0000
Sample Amount:              :     1.00000  [ng/ul]   (not used in calc.)
Do not use Multiplier & Dilution Factor with ISTDs


Signal 1: DAD1 B, Sig=214,4 Ref=off
```

Peak #	RetTime [min]	Type	Width [min]	Area [mAU*s]	Height [mAU]	Area %
1	6.471	MM	0.2324	1.42898e4	1024.70313	50.8096
2	7.288	MM	0.2676	1.38344e4	861.54517	49.1904

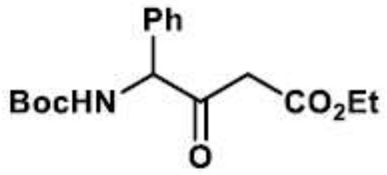

Data File C:\Chem32\1\Data\Srinivas\IT-4713 Racemic 2022-02-01 12-24-29.D
Sample Name: IT-4713 Racemic

Totals : 2.81242e4 1886.24829

Signal 2: DAD1 C, Sig=254,4 Ref=off

Peak #	RetTime [min]	Type	Width [min]	Area [mAU*s]	Height [mAU]	Area %
1	6.469	MM	0.2270	2451.50684	180.02174	50.9596
2	7.284	MM	0.2650	2359.18286	148.37228	49.0404

Totals : 4810.68970 328.39403

*** End of Report ***

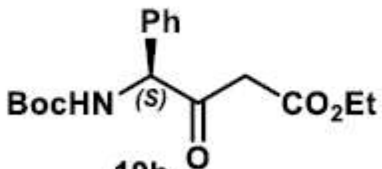

19b

Data File C:\Chem32\1\Data\Srinivas\IT-4723 S 2022-02-01 12-24-42.D
Sample Name: IT-4723 S

Acq. Operator : SYSTEM
Sample Operator : SYSTEM
Acq. Instrument : CHIRAL Location : 3
Injection Date : 2/1/2022 1:13:39 PM
Inj Volume : 5.000 µl
Acq. Method : C:\CHEM32\1\METHODS\IA_nHep_Ethanol_Custom.M
Last changed : 2/1/2022 12:30:24 PM by SYSTEM
Analysis Method : C:\Chem32\1\Methods\Method_develop_Chiral_IF_nHep_Ethanol.M
Last changed : 3/18/2021 4:41:28 PM by SYSTEM
Method Info : method development for normal phase chiral separations ChiralPak IF with n-Heptane and Ethanol

Additional Info : Peak(s) manually integrated

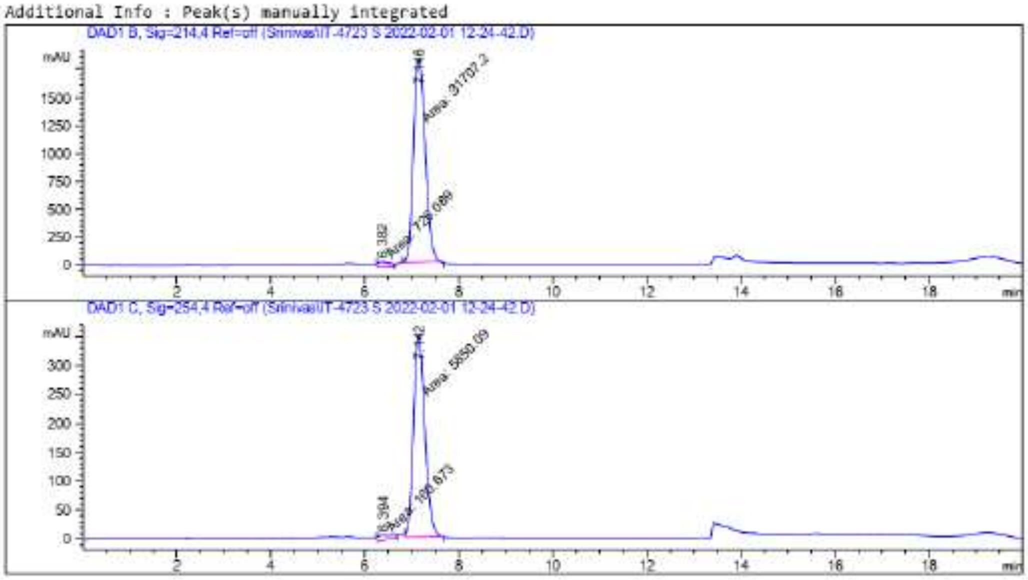

Area Percent Report

Sorted By : Signal
Multiplier : 1.0000
Dilution : 1.0000
Sample Amount: : 1.00000 [ng/ul] (not used in calc.)
Do not use Multiplier & Dilution Factor with ISTDs

Signal 1: DAD1 B, Sig=214,4 Ref=off

Peak #	RetTime [min]	Type	Width [min]	Area [mAU*s]	Height [mAU]	Area %
1	6.382	MM	0.2856	726.08911	42.37216	2.2387
2	7.146	MM	0.2922	3.17072e4	1808.41626	97.7613

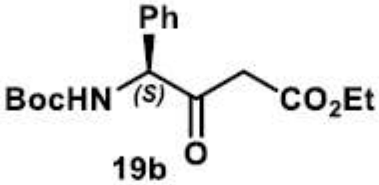

19b

Data File C:\Chem32\1\Data\Srinivas\IT-4723 S 2022-02-01 12-24-42.D
Sample Name: IT-4723 S

Totals : 3.24333e4 1850.78842

Signal 2: DAD1 C, Sig=254,4 Ref=off

Peak #	RetTime [min]	Type	Width [min]	Area [mAU*s]	Height [mAU]	Area %
1	6.394	MM	0.3181	160.67343	8.41785	2.6731
2	7.142	MM	0.2792	5850.09180	349.22180	97.3269

Totals : 6010.76523 357.63965

*** End of Report ***

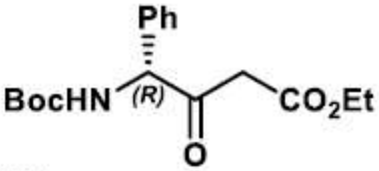

Data File C:\Chem32\1\Data\Srinivas\IT-4747 R 2022-02-01 12-30-33.D
Sample Name: IT-4747 R

```
=====================================================================
Acq. Operator   : SYSTEM
Sample Operator : SYSTEM
Acq. Instrument : CHIRAL                          Location :   4
Injection Date  : 2/1/2022 1:56:02 PM
                                               Inj Volume : 5.000 µl
Acq. Method     : C:\CHEM32\1\METHODS\IA_nHep_Ethanol_Custom.M
Last changed    : 2/1/2022 12:30:24 PM by SYSTEM
Analysis Method : C:\Chem32\1\Methods\Method_develop_Chiral_IF_nHep_Ethanol.M
Last changed    : 3/18/2021 4:41:28 PM by SYSTEM
Method Info     : method development for normal phase chiral separations ChiralPak IF with n-
                  Heptane and Ethanol

Additional Info : Peak(s) manually integrated
```

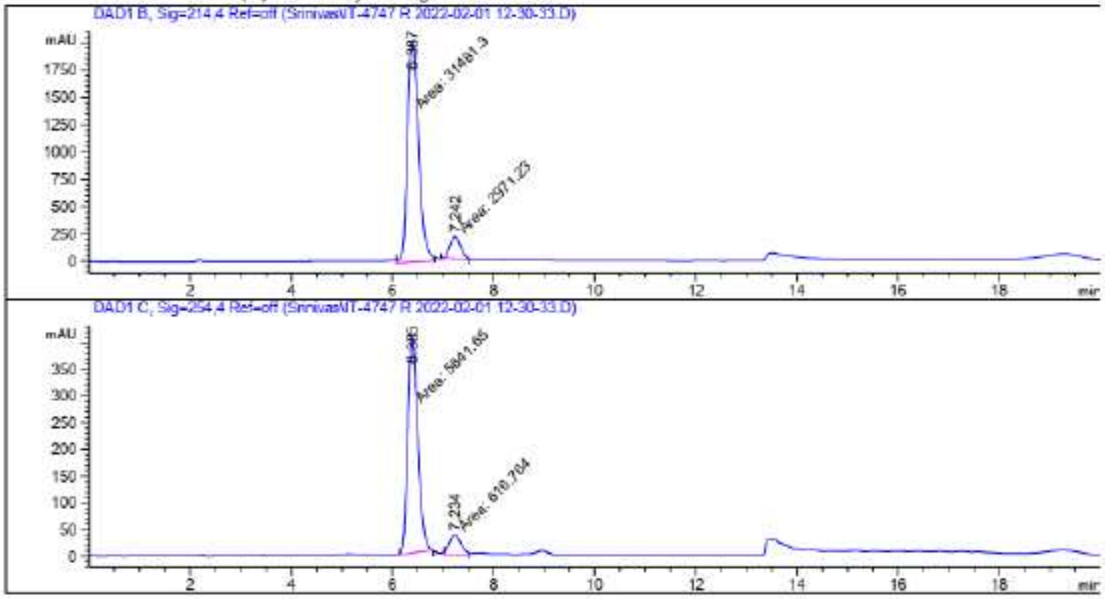


```
=====================================================================
                     Area Percent Report
=====================================================================

Sorted By             :      Signal
Multiplier            :      1.0000
Dilution              :      1.0000
Sample Amount:               :     1.00000  [ng/ul]   (not used in calc.)
Do not use Multiplier & Dilution Factor with ISTDs


Signal 1: DAD1 B, Sig=214,4 Ref=off
```

Peak #	RetTime [min]	Type	Width [min]	Area [mAU*s]	Height [mAU]	Area %
1	6.387	MM	0.2606	3.14813e4	2013.11328	91.3759
2	7.242	MM	0.2471	2971.23193	200.36804	8.6241

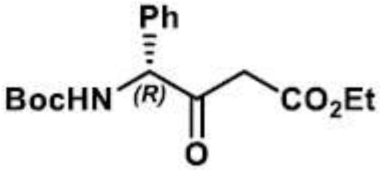

Data File C:\Chem32\1\Data\Srinivas\IT-4747 R 2022-02-01 12-30-33.D
Sample Name: IT-4747 R

Totals : 3.44526e4 2213.48132

Signal 2: DAD1 C, Sig=254,4 Ref=off

Peak #	RetTime [min]	Type	Width [min]	Area [mAU*s]	Height [mAU]	Area %
1	6.385	MM	0.2315	5641.65381	406.22049	90.1451
2	7.234	MM	0.2674	616.76367	38.43676	9.8549

Totals : 6258.41748 444.65725

*** End of Report ***

Data File C:\Chem32\1\Data\Srinivas\4796 Upper and Lower 2022-05-12 14-51-01\002-0201.D
Sample Name: Upper Racemic

===

Acq. Operator : SYSTEM Seq. Line : 2
Acq. Instrument : CHIRAL Location : 2
Injection Date : 5/12/2022 3:09:11 PM Inj : 1
Inj Volume : 10.000 µl
Method : C:\Chem32\1\Data\Srinivas\4796 Upper and Lower 2022-05-12 14-51-01\FAST_Chiral_Scout_IF_nHep_Ethanol.M (Sequence Method)
Last changed : 5/12/2022 2:51:01 PM by SYSTEM
Method Info : Scouting method for normal phase chiral separations ChiralPak IF with n-Heptane and Ethanol

Additional Info : Peak(s) manually integrated

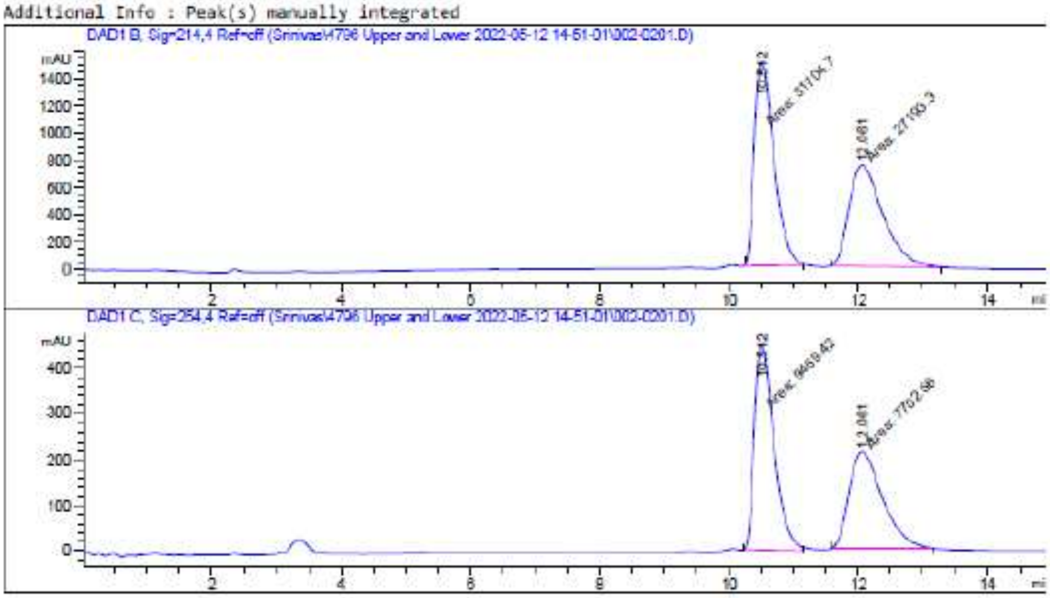

===

Area Percent Report

===

Sorted By : Signal
Multiplier : 1.0000
Dilution : 1.0000
Do not use Multiplier & Dilution Factor with ISTDs

Signal 1: DAD1 B, Sig=214,4 Ref=off

Peak #	RetTime [min]	Type	Width [min]	Area [mAU*s]	Height [mAU]	Area %
1	10.512	MM	0.3518	3.17047e4	1501.98840	53.8298
2	12.061	MM	0.6105	2.71933e4	742.32043	46.1702

Totals : 5.88980e4 2244.30884

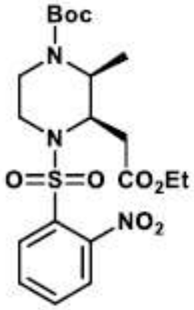

(±)

Data File C:\Chem32\1\Data\Srinivas\4796 Upper and Lower 2022-05-12 14-51-01\002-0201.D
Sample Name: Upper Racemic

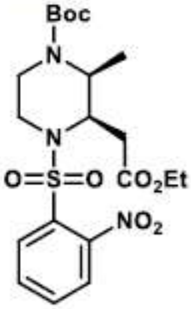

(±)

Signal 2: DAD1 C, Sig=254,4 Ref=off

Peak #	RetTime [min]	Type	Width [min]	Area [mAU*s]	Height [mAU]	Area %
1	10.512	MM	0.3495	9469.42383	451.55734	55.1446
2	12.061	MM	0.6012	7702.55957	213.53856	44.8554
Totals :				1.71720e4	665.09590	

*** End of Report ***

Data File C:\Chem32\1\Data\Srinivas\4796 Upper and Lower 2022-05-12 14-51-01\003-0301.D
Sample Name: 4796 Upper

===

Acq. Operator : SYSTEM Seq. Line : 3
Acq. Instrument : CHIRAL Location : 3
Injection Date : 5/12/2022 3:25:22 PM Inj : 1
Inj Volume : 10.000 µl
Method : C:\Chem32\1\Data\Srinivas\4796 Upper and Lower 2022-05-12 14-51-01\FAST_Chiral_Scout_IF_nHep_Ethanol.M (Sequence Method)
Last changed : 5/12/2022 2:51:01 PM by SYSTEM
Method Info : Scouting method for normal phase chiral separations ChiralPak IF with n-Heptane and Ethanol

Additional Info : Peak(s) manually integrated

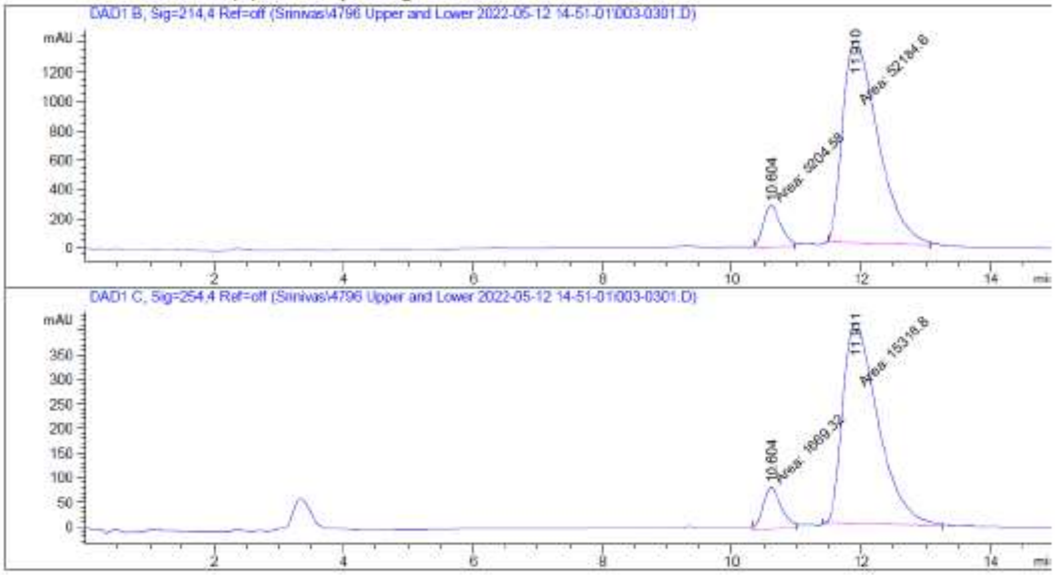

===

Area Percent Report

===

Sorted By : Signal
Multiplier : 1.0000
Dilution : 1.0000
Do not use Multiplier & Dilution Factor with ISTDs

Signal 1: DAD1 B, Sig=214,4 Ref=off

Peak #	RetTime [min]	Type	Width [min]	Area [mAU*s]	Height [mAU]	Area %
1	10.604	MM	0.3087	5204.57568	280.98712	9.0689
2	11.910	MM	0.6312	5.21846e4	1377.94214	90.9311

Totals : 5.73891e4 1658.92926

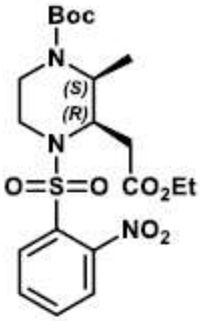

S5

Data File C:\Chem32\1\Data\Srinivas\4796 Upper and Lower 2022-05-12 14-51-01\003-0301.D
Sample Name: 4796 Upper

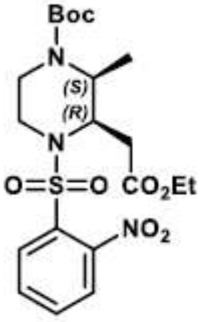

S5

Signal 2: DAD1 C, Sig=254,4 Ref=off

Peak #	RetTime [min]	Type	Width [min]	Area [mAU*s]	Height [mAU]	Area %
1	10.604	MM	0.3263	1669.32361	85.27359	9.8264
2	11.911	MM	0.6264	1.53188e4	407.59848	90.1736
Totals :				1.69881e4	492.87207	

*** End of Report ***

Data File C:\Chem32\1\Data\Srinivas\4796 Upper and Lower 2022-05-12 14-51-01\004-0501.D
Sample Name: Lower Racemic

===

Acq. Operator	: SYSTEM	Seq. Line :	5
Acq. Instrument	: CHIRAL	Location :	4
Injection Date	: 5/12/2022 3:57:49 PM	Inj :	1
		Inj Volume :	10.000 µl

Method : C:\Chem32\1\Data\Srinivas\4796 Upper and Lower 2022-05-12 14-51-01\FAST_Chiral_Scout_IF_nHep_Ethanol.M (Sequence Method)
Last changed : 5/12/2022 2:51:01 PM by SYSTEM
Method Info : Scouting method for normal phase chiral separations ChiralPak IF with n-Heptane and Ethanol

Additional Info : Peak(s) manually integrated

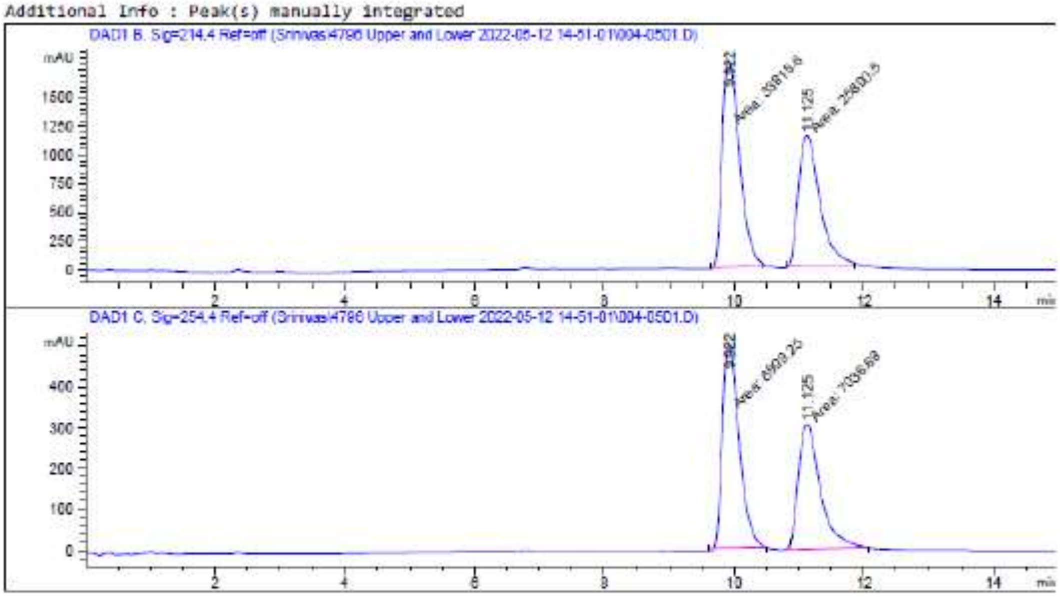

===
Area Percent Report
===

Sorted By : Signal
Multiplier : 1.0000
Dilution : 1.0000
Do not use Multiplier & Dilution Factor with ISTDs

Signal 1: DAD1 B, Sig=214,4 Ref=off

Peak #	RetTime [min]	Type	Width [min]	Area [mAU*s]	Height [mAU]	Area %
1	9.922	MM	0.3119	3.38156e4	1806.72754	56.7222
2	11.125	MM	0.3794	2.58005e4	1133.49927	43.2778
Totals :				5.96161e4	2940.22681	

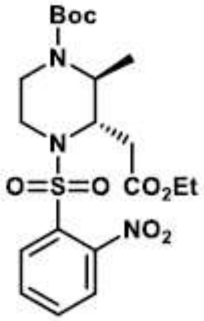

(±)

Data File C:\Chem32\1\Data\Srinivas\4796 Upper and Lower 2022-05-12 14-51-01\004-0501.D
Sample Name: Lower Racemic

Signal 2: DAD1 C, Sig=254,4 Ref=off

Peak #	RetTime [min]	Type	Width [min]	Area [mAU*s]	Height [mAU]	Area %
1	9.922	MM	0.3000	8999.25293	499.96991	56.1193
2	11.125	MM	0.3837	7036.67822	305.66632	43.8807

Totals : 1.60359e4 805.63623

*** End of Report ***

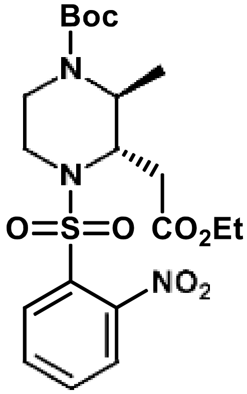

(±)

Data File C:\Chem32\1\Data\Srinivas\4796 Upper and Lower 2022-05-12 14-51-01\005-0601.D
Sample Name: 4796 Lower

===

Acq. Operator : SYSTEM Seq. Line : 6
Acq. Instrument : CHIRAL Location : 5
Injection Date : 5/12/2022 4:14:00 PM Inj : 1
Inj Volume : 10.000 µl
Method : C:\Chem32\1\Data\Srinivas\4796 Upper and Lower 2022-05-12 14-51-01\FAST_Chiral_Scout_IF_nHep_Ethanol.M (Sequence Method)
Last changed : 5/12/2022 2:51:01 PM by SYSTEM
Method Info : Scouting method for normal phase chiral separations ChiralPak IF with n-Heptane and Ethanol

Additional Info : Peak(s) manually integrated

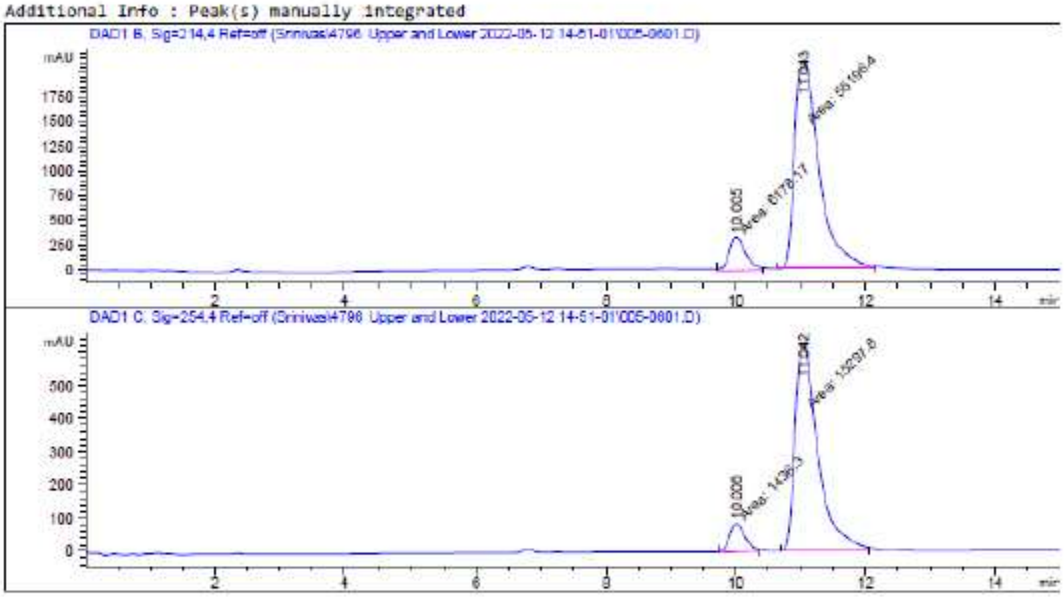

===

Area Percent Report

===

Sorted By : Signal
Multiplier : 1.0000
Dilution : 1.0000
Do not use Multiplier & Dilution Factor with ISTDs

Signal 1: DAD1 B, Sig=214,4 Ref=off

Peak #	RetTime [min]	Type	Width [min]	Area [mAU*s]	Height [mAU]	Area %
1	10.005	MM	0.3009	6178.16748	342.18903	10.0663
2	11.043	MM	0.4382	5.51964e4	2099.14819	89.9337

Totals : 6.13746e4 2441.33722

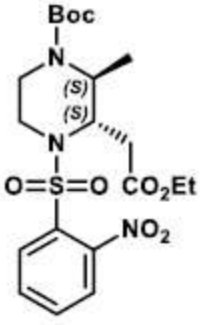

S6

Data File C:\Chem32\1\Data\Srinivas\4796 Upper and Lower 2022-05-12 14-51-01\005-0601.D
Sample Name: 4796 Lower

Signal 2: DAD1 C, Sig=254,4 Ref=off

Peak #	RetTime [min]	Type	Width [min]	Area [mAU*s]	Height [mAU]	Area %
1	10.006	MM	0.2793	1436.29565	85.71114	8.5830
2	11.042	MM	0.4102	1.52978e4	621.56360	91.4170
Totals :				1.67341e4	707.27473	

===

*** End of Report ***

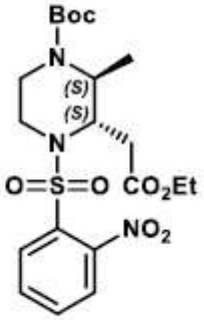

S6
